# Population genomics of Nigerian goat breeds and neighbouring populations in the West Africa–Cameroon transboundary livestock corridor

**DOI:** 10.1371/journal.pone.0354294

**Published:** 2026-07-27

**Authors:** Oludayo Michael Akinsola, Olusegun Olaniyi Adeniyi, Oladeji Bamidele, Oluyinka Opoola, David Oludare Omoniwa, Abdulmojeed Yakubu, Chitra Ramasamy, Malarmathi Muthusamy, Aranganoor Kannan Thiruvenkadan, Abdulraheem Arome Musa

**Affiliations:** 1 Department of Theriogenology and Production, Faculty of Veterinary Medicine, University of Jos, Jos, Nigeria; 2 Department of Animal Genetics and Breeding, Veterinary College and Research Institute, TANUVAS, Namakkal, India; 3 Institute of Animal Breeding and Genetics, Justus Liebig University, Giessen, Germany; 4 Department of Biological Sciences, Faculty of Sciences, Kings University, Odeomu, Nigeria; 5 People, Policies and Institutions (PPI) Program, International Livestock Research Institute, Nairobi, Kenya; 6 Centre for Tropical Livestock Genetics and Health (CTLGH), Roslin Institute, University of Edinburgh, Easter Bush Campus, Edinburgh, United Kingdom; 7 Global Academy of Agriculture and Food Systems (GAAFS) and the Royal (Dick) School of Veterinary Studies (RDSVS), University of Edinburgh, Easter Bush Campus, Edinburgh, United Kingdom; 8 Department of Veterinary Medicine, Faculty of Veterinary Medicine, University of Jos, Jos, Nigeria; 9 Department of Animal Science, Faculty of Agriculture, Nasarawa State University, Keffi, Nigeria; 10 College of Poultry Production and Management, TANUVAS, Hosur, India; 11 Research Institute for Farm Animal Biology (FBN), Dummerstorf, Germany; Federal University of Agriculture, NIGERIA

## Abstract

Indigenous goats in Nigeria and neighbouring countries support livelihoods across forest–savanna–Sahel environments, yet genomic structure, connectivity history, and adaptive signals are rarely investigated in a single corridor-scale transboundary framework. We analysed three Nigerian populations (Sahel, Red Sokoto/Maradi, and West African Dwarf; WAD) and seven neighbouring populations from Burkina Faso, Mali, and Cameroon using 46,431 autosomal markers from 209 unrelated animals (with a South Asian outgroup where needed). We tested whether recurrent vernacular labels map onto shared genomic backgrounds across borders, reconstructed time-layered connectivity, quantified demographic contraction and inbreeding, and prioritised candidate adaptive regions using a structure-aware approach. Model-based ancestry and principal component analysis supported three transboundary genomic backgrounds: (i) a Sahel–Sudan background spanning Nigeria, Burkina Faso, and Mali; (ii) a southern Djallonké/WAD background spanning Nigeria, Burkina Faso, and Mali; and (iii) a distinct Cameroon dwarf lineage, with Guéra representing a drifted subgroup within the Sahel–Sudan background. Admixture-timing analysis, interpreted as approximate dates inferred from linkage-disequilibrium decay, suggested very recent cross-border involving Nigerian Sahel goats (~30–40 years under the assumed generation interval), superimposed on older Sahelian–dwarf exchange (~160–1,000 years). Effective population size declined from ~1,400–2,700 at ~960 generations ago to ~40–111 at 13 generations ago. Runs of homozygosity indicated low-to-moderate genomic inbreeding (0.004–0.040), with long segments (>8 Mb) most pronounced in Guéra and Red Sokoto/Maradi. A multi-statistic composite selection scan identified 53 candidate windows. Enrichment highlighted adhesion and translation quality-control themes in the Djallonké/WAD background background, neuronal/neuroendocrine terms in Guéra, and olfactory transduction in the Sahel–Sudan background. These results define transboundary genomic backgrounds rather than country-bounded “breeds” and provide background-specific hypotheses that can be validated in resilience-oriented breeding under ongoing mobility.

## Introduction

Goats (*Capra hircus*), domesticated around 11,000 years before present [1], were introduced into Africa from Southwest Asia and subsequently diversified under regional movement and admixture [2,3]. They are central to smallholder and pastoral livelihoods across the West Africa–Cameroon region, where production spans a steep forest–savanna–Sahel gradient with pronounced heterogeneity in heat load, disease challenge, and seasonal feed scarcity. Goats supply meat, milk and skins, and they serve as a liquid asset for household risk management; ownership and use are often shaped by gendered roles and intra-household decision-making [4]. Despite their importance, productivity per animal remains modest in these production systems, and chronic feed deficits are repeatedly identified as a major constraint [5]. In this context, genetic improvement and conservation are most likely to succeed when framed as complementary to management interventions and grounded in an accurate understanding of population structure, connectivity, and diversity at the spatial scales where animals are exchanged [6,7].

A practical but seldom tested premise underpins how goats are discussed and managed across the region, as the same vernacular “breed names” recur across multiple African countries, especially along the Sahel belt and within the humid southern belt. Labels such as “Sahel” and “West African Dwarf/Djallonké” are used routinely in national statistics, markets, and extension, implying discrete units that can be monitored, conserved, or improved. However, these labels are also embedded in a transboundary exchange system that links Sahelian pastoral zones, savanna trade corridors, and humid-belt smallholder systems, consistent with evidence that drought adaptation, trade histories and contemporary transhumance continue to structure livestock mobility and cross-border circulation in West Africa [8–10]. The key question is therefore whether these labels track coherent genomic backgrounds or socially stable phenotypes maintained under recurrent movement and admixture.

Nigeria mirrors this broader naming system and is commonly described as comprising three main indigenous breed types aligned with eco-climatic zones: Sahel goats in the arid and semi-arid north, Red Sokoto/Maradi goats in the savanna belt, and West African Dwarf (WAD; Djallonké-type) goats in the humid tsetse belt [11,12]. These labels capture broad phenotypic patterns and production ecologies, including well-recognised differences in body size and adaptive performance. WAD goats, in particular, have been reported to show resistance/resilience to gastrointestinal nematodes and trypanotolerance, supported by field and immunological evidence [13–15]. However, because goats circulate through trade networks and transhumance, vernacular names and national “breeds” may not correspond to discrete genomic units; they may instead denote phenotypes that persist despite recurrent exchange and admixture.

Population genetic studies of West African goats have historically reported high within-population diversity and low to moderate differentiation, consistent with substantial connectivity among Sahelian and Djallonké-type populations [16,17]. The AdaptMap project extended this perspective using global 50K single-nucleotide ploymorphism (SNP) data, revealing broad continental structure and extensive admixture within Africa [2]. Subsequent SNP-chip analyses in African goats have characterised diversity, inbreeding, and demographic history, including genome-wide evidence for the differentiation of Cameroon goats within broader African and Asian comparative panels and evidence for recent effective population size contraction in multiple settings [3,18,19]. In parallel, genome-wide scans have identified candidate loci and pathways potentially related to health and environmental adaptation in small ruminants, although interpretation is often constrained by the spatial scale and sampling frame used to define populations [20].

Despite this progress, Nigerian goats and their neighbouring populations remain insufficiently addressed within a framework that aligns with how animals are exchanged and how management decisions are made. Three gaps are particularly relevant. First, although recurrent breed names are used across borders, whether these labels map to shared genomic backgrounds or instead reflect phenotypic convergence under similar environments amid ongoing exchange has seldom been evaluated using a single, explicitly transboundary sampling design. Second, Nigeria’s three major breed labels are typically discussed as national entities. However, their genomic interpretation depends on corridor-scale connectivity with cognate populations in Burkina Faso, Mali, and Cameroon, where movement along the Sahel belt and through savanna–forest interfaces can generate repeated admixture. Third, there remains a need to integrate inference on structure, connectivity chronology, demography, and adaptive signatures within a single corridor-scale design that can support conservation units and resilient breeding strategies under realistic exchange systems without relying on assumptions of closed national breeds. In this study, “transboundary genomic backgrounds” refers to broad genomic affinities shared by populations sampled across national borders. We use the term to avoid treating national breed labels as closed units and to reflect the possibility that ancestry, movement and admixture may connect populations with similar vernacular or ecotype labels across the corridor.

Here, we address these gaps by analysing Nigerian Sahel, Red Sokoto/Maradi and WAD goats alongside neighbouring Sahelian and Djallonké/WAD-type populations from Burkina Faso and Mali and a Cameroon dwarf population within a West Africa–Cameroon transboundary livestock corridor framework, using the public AdaptMap GoatSNP50 resource [2]. Our objectives are to (i) test whether recurrent vernacular labels correspond to coherent genomic backgrounds across borders, (ii) quantify corridor-scale connectivity and admixture chronology that can reconcile ecotype-associated structure with ongoing exchange, (iii) characterise demographic pressures and inbreeding patterns relevant to management, and (iv) identify candidate adaptive signals in a structure-informed way that connects candidate regions to corridor-defined genomic backgrounds and prioritises biologically interpretable, testable hypotheses rather than producing generic gene lists. By placing Nigerian breeds within transboundary genomic backgrounds, this study provides a corridor-scale genomic baseline intended to inform conservation planning and resilient improvement strategies that align with how goats are actually exchanged and managed.

## Materials and methods

### Ethics statement

This study re-analyses genotypes from the public AdaptMap dataset; no new animal handling was performed, and no new ethical approval was required (data provenance and accession are detailed below).

### Data provenance and study populations

We analysed SNP genotypes from the public AdaptMap GoatSNP50 working panel, generated on the Illumina CaprineSNP50 BeadChip and mapped to the *Capra hircus* ARS1 reference assembly, as described in the AdaptMap data description [2]. From this resource, we assembled a dataset of 216 goats from 11 populations spanning the humid-forest to Sahel eco-climatic gradient across West Africa and Cameroon, and included Koh-e-Sulmani (PK_KES; n = 14) from Pakistan as a South Asian outgroup for analyses that require rooting or external contrast. The study populations comprised: Burkina Faso Djallonké (BF_DJA, n = 12) and Sahel (BF_SHL, n = 15); Cameroon WAD (CM_WAD, n = 34); Mali Guéra (ML_GUE, n = 25), Maure (ML_MAU, n = 14), Naine (ML_NAI, n = 17), and Targui (ML_TAR, n = 22); and Nigeria Red Sokoto/Maradi (NG_RSK, n = 21), Sahel (NG_SHL, n = 21), and WAD (NG_WAD, n = 21). For consistency with our nomenclature, samples labelled “BF_SAH” in AdaptMap were harmonised to “BF_SHL” prior to analysis. These populations define a West Africa–Cameroon transboundary livestock corridor sampling frame within the AdaptMap GoatSNP50 panel [2].

### Genotype management and quality control

All data processing was performed using *PLINK* v1.9 [21]. The merged dataset initially comprised 216 individuals and 53,347 SNPs. Restricting the data to autosomal markers on chromosomes 1–29 (*PLINK* --chr-set 29 --autosome) yielded 49,953 autosomal SNPs. No individual failed the missingness screen (--mind 0.10, i.e., ≤10% missing genotypes), so 216/216 samples were retained. Subsequent variant-level quality control (QC) removed 1,517 SNPs with call rate <95% (--geno 0.05) and 2,005 SNPs with minor allele frequency (MAF) <0.05 (--maf 0.05), leaving 46,431 autosomal SNPs for downstream analyses. To reduce bias from close kinship, relatedness pruning was performed within each population using linkage disequilibrium (LD)-pruned SNPs (as described below) and variance-standardised genomic relationship matrix with --rel-cutoff 0.35; one from each related pair exceeding the threshold was removed, yielding a final panel of 209 individuals used for population-genetic analyses. Unless stated otherwise, all downstream analyses use this unrelated set. For analyses requiring unlinked markers (e.g., principal component analysis (PCA) and admixture analyses), LD pruning used --indep-pairwise 50 5 0.2, producing 39,848 SNPs.

### Population structure

Using the unrelated, LD-pruned dataset, we inferred individual ancestry with *ADMIXTURE* (v1.3) under unsupervised models for K=1−7 ancestral clusters and selected the best-supported K based on the minimum using cross-validation (CV) error [22,23]. The individual-by-cluster ancestry proportion matrices were visualised in R using *pophelper* for barplots [24]. We ran PCA [25,26] in PLINK on the same pruned marker set. To focus interpretation on the corridor itself, we report PCA results for both the full panel (11 populations, including PK_KES) and a restricted panel excluding the South-Asian outgroup (10 African corridor populations: Burkina Faso, Mali, Nigeria, and Cameroon). PCA plots were generated with *ggplot2* v3.5.0 [27].

### Genetic differentiation

We estimated pairwise genetic differentiation as Weir & Cockerham’s fixation index ($F_{ST}$) [28] per SNP for all breed pairs in *PLINK* (--fst --within) [21] using the dense unrelated post-QC marker set (209 individuals; 46,431 autosomal SNPs), not the LD-pruned marker set. We computed $F_{ST}$ per SNP. Negative per-SNP estimates were set to zero prior to averaging, as negatives reflect sampling variance. Pairwise mean $F_{ST}$ values were assembled into a matrix and visualised as a heat map using R package *ggplot2* v3.5.0 [27].

### Historical gene flow

We modelled population relationships with *TreeMix* v1.13 [29] using per-population allele counts derived from the unrelated post-QC panel (209 individuals; 46,431 autosomal SNPs). For each breed, *PLINK* allele counts (--freq counts) were harmonised to a common reference allele across populations. Trees were rooted on PK_KES and fitted with m=0−6 migration edges. To retain marker density, we did not further LD-prune; instead, 500-SNP block jackknifing provided robust standard errors. Model adequacy and the preferred m were determined from improvements in likelihood/variance explained and reduced structured residual covariance in *TreeMix* diagnostics [29].

### Dating of admixture events

Admixture timing was inferred with *ALDER* v1.03, which estimates time since admixture from the exponential decay of weighted LD as a function of genetic distance [30]. Estimates in generations were converted to years assuming a 3.7-year generation interval and a uniform recombination rate of 1 cM/Mb in the absence of a high-resolution caprine map. The unpruned post-QC autosomal panel (46,431 SNPs) was restricted to the unrelated sample set (209 animals) and converted to EIGENSTRAT format with *convertf* [25,26]. For each Nigerian target (NG_SHL, NG_RSK, NG_WAD), we ran ALDER with the seven corridor populations (BF_SHL, BF_DJA, CM_WAD, ML_GUE, ML_MAU, ML_NAI, ML_TAR) as reference populations and extracted one-reference weighted-LD fits for each target–reference combination. We interpret significant one-reference curves as dating admixture-related LD in the target using the named reference as a proxy for a contributing ancestry component, recognising that one-reference fits do not constitute a standalone formal admixture test. To suppress short-range background LD, we fitted *ALDER* one-reference curves across a grid of minimum distances $d_{min}\in \left\{\text{0}.\text{3},\text{0}.\text{4},\text{0}.\text{5},\text{0}.\text{6},\text{0}.\text{7}\right\}$ cM and used chromosome-jackknife standard errors; statistical support followed the ALDER convention (LD-decay Z ≥ 2.0). Target–reference combinations were classified as stable when they remained significant at ≥3 adjacent $d_{min}$ values with coefficient of variation ≤0.25 across those dates, consistent when they were significant at ≥2 adjacent 𝑑*_min_* values, fragile when supported at only one or non-robust start distance, and none when no significant LD-decay fit was detected. Because one-reference ALDER fits can be affected by background LD generated by demography, dates are interpreted as admixture-related LD-decay proxy dates and evaluated together with the population-structure and gene-flow results.

### Demographic history

#### Effective population size.

We inferred the recent effective population size ($N_{e}$) from LD using *SNeP* v1.1 [31]. For each breed, analyses were run on the QC-filtered autosomal dataset (46,431 SNPs) for the same 209 unrelated animals used in the population-structure analyses, with no additional SNP pruning beyond QC, estimating pairwise $r^{\text{2}}$ in 50-kb bins across 50 kb–4 Mb with a minimum $r^{\text{2}}$ of 0.01. We fitted the Sved–Feldman expectation [32] with mutation correction α = 2 [33] under a recombination rate of $\text{1}\times {\text{1}\text{0}}^{-\text{8}}$ per bp per generation to obtain $N_{e}$ trajectories. Curves are interpreted only over distance ranges with adequate SNP support, acknowledging the sensitivity of LD-based $N_{e}$ to sample size and array density [34,35].

#### Runs of homozygosity (ROH) and genomic inbreeding.

Runs of homozygosity were identified on autosomes using *PLINK* in a cohort-level dataset restricted to the ten focal African corridor populations (excluding PK_KES). For ROH-specific quality control, individuals with >10% missing genotypes and SNPs with >5% missingness were removed, while no minor-allele-frequency or Hardy–Weinberg-equilibrium filters were applied to avoid bias against rare variants within ROH. Near-identical genotype duplicates (pairwise identity-by-descent π̂ ≥ 0.95) were excluded based on genome-wide estimates, and population-specific subsets were then generated from the remaining animals. ROH were called in each population using PLINK’s sliding-window algorithm [36], with a minimum segment length of 1 Mb, a minimum SNP density of 1 SNP per 150 kb, a maximum gap of 1 Mb between consecutive SNPs, and allowing up to 2 missing calls per scanning window and 0 heterozygotes. Up to two missing calls per scanning window were allowed to avoid fragmenting ROH because of isolated missing genotypes, whereas heterozygous calls were not permitted to keep ROH detection conservative in medium-density SNP-chip data. The minimum number of SNPs required to define an ROH(L) was determined following [37], using observed heterozygosity and sample size, so that the expected probability of observing ≥1 ROH by chance was ≤0.05. The resulting threshold was constrained to l ≥ 30 SNPs to stabilise ROH calling with 50K SNP density. For each individual, genomic inbreeding ($F_{ROH}$) was calculated as the total length of autosomal ROH divided by the length of the autosomal genome covered by SNPs, and ROH were partitioned into 1–4, 4–6, 6–8 and ≥8 Mb length classes to separate signals of more ancient from more recent inbreeding [38,39].

### Selective-sweep detection and functional annotation of candidate regions

Per-breed datasets were derived by subsetting the filtered binary *PLINK* files to breed-specific sample lists, and chromosomes were split to facilitate downstream processing. Variant Call Format (VCF) files were exported per chromosome, checked with *BCFtools* v1.9 [40] to remove duplicate records and retain biallelic SNPs, then block-gzipped and indexed. Phasing was performed with *Beagle* v5.1 [41,42]. These steps yielded a consistent, phased autosomal panel of biallelic SNPs suitable for haplotype-based statistics in each population.

#### Per-statistic computations.

We combined five complementary statistics per breed for the de-correlated composite of multiple signals (DCMS):

**Integrated haplotype score (**iHS**):** Computed within population using *rehh* v 3.2.2 [43,44], ingesting phased VCFs by chromosome with no ancestral polarisation (polarize_vcf = FALSE). Extended haplotype homozygosity was scanned with an integration cap of 500 kb (maxgap=$\text{5}\times {\text{1}\text{0}}^{\text{5}}$). Genome-wide iHS values were derived with *ihh2ihs* function (min MAF = 0.05; frequency bins = 0.1) and transformed to |iHS| for DCMS.

**Cross-population extended haplotype homozygosity (XP-EHH):** We computed XP-EHH in *rehh* from the same phased, per-chromosome VCFs (chr 1–29), again without ancestral polarisation. For each chromosome, EHH profiles were scanned in each population and contrasted to obtain XP-EHH, which was then Z-standardised genome-wide within each target–reference comparison and averaged on the shared non-overlapping 500-kb window grid. Where a target population was evaluated against multiple references, the resulting window-level Z-scores were combined across the pre-defined reference set using Stouffer’s method, yielding a single window-level aggregate per target for DCMS. Reference sets were specified a priori to reflect the corridor contrasts: NG_SHL against BF_SHL, ML_MAU and ML_TAR; NG_RSK against NG_SHL and BF_SHL; NG_WAD against BF_DJA, CM_WAD and ML_NAI; BF_SHL against ML_TAR and ML_MAU; BF_DJA against CM_WAD and NG_WAD; ML_MAU against ML_TAR and BF_SHL; ML_TAR against ML_MAU and BF_SHL; ML_NAI against BF_DJA and CM_WAD; ML_GUE against ML_MAU and BF_SHL; and CM_WAD against BF_DJA and NG_WAD.

**Number of segregating sites by length (**nSL**):** The nSL was computed with *selscan* v.2.1 [45,46] on phased VCFs for each chromosome. Raw nSL scores were normalised within allele-frequency bins (0.05 to 0.95 in 0.05 increments) using the frequency reported by *selscan*; no ancestral polarisation was applied. Bin-wise Z-normalisation was performed within each frequency bin; where this could not be computed (e.g., due to insufficient variation within a bin), bins were instead normalised using a global Z-normalisation across sites.

**Haplotype homozygosity (**H12**):** Computed following the implementation of Garud et al. [47] (25-SNP windows, 1-SNP step); windows with <3 observed haplotypes were discarded. The H12 values were assigned to window centres for aggregation.

**Pooled heterozygosity (**${ZH}_{p}$**):** Within fixed 500 kb windows on a shared genome-wide grid (anchored at base 1), we summed the major-allele count ($n_{maj}$) and minor-allele counts ($n_{min}$) per SNP from breed-specific allele-count files and computed pooled heterozygosity per window as $H_{p}=\frac{\text{2}\left(\sum n_{maj}\right)\left(\sum n_{min}\right)}{{\left(\sum n_{maj+}\sum n_{min}\right)}^{\text{2}}}$ [48]. Windows with <10 SNPs were excluded. $H_{p}$ was Z-standardised within breed (${ZH}_{p}$); low ${ZH}_{p}$ indicates local dips in diversity.

All five statistics were aggregated into a common set of non-overlapping 500-kb windows per autosome. For iHS, nSL, H12 and XP-EHH, we averaged all values whose genomic positions fell within a window; ${ZH}_{p}$ already matched the window grid. Haplotype-based statistics were emphasised because recent or incomplete sweeps can generate extended haplotype homozygosity even when between-population allele-frequency differentiation remains modest. To include a complementary diversity-based signal, ${ZH}_{p}$ was also included to identify windows with unusually low pooled heterozygosity.

#### Empirical p-values, de-correlation and DCMS.

Within each breed, window-level values were converted to empirical, rank-based p-values using the fractional-rank approach implemented in R package *MINOTAUR* [49], with tail directions defined a priori: right-tailed for |iHS|, XP-EHH, and H12, two-tailed for nSL, left-tailed for ${ZH}_{p}$. To account for redundancy among statistics, we estimated a robust covariance matrix (M-estimator) and computed DCMS scores by summing logit-transformed p-values with correlation-aware weights as described by Ma et al. [50] and implemented in *MINOTAUR*. We converted DCMS scores to upper-tail p-values by robust normal modelling of the genome-wide score distribution. We controlled the false discovery rate (FDR) at 5% [51]. Windows with q<0.05 were declared DCMS outliers within breed.

#### Functional annotation of candidate regions.

Significant DCMS windows (q < 0.05) were queried against the Ensembl *Capra hircus* ARS1 gene annotation via the Representational State Transfer (REST) application programming interface [52] to identify Ensembl genes overlapping each candidate window. Gene symbols, gene biotypes, and descriptive annotations were harmonised using *biomaRt* v2.60.1 [53].

#### Gene ontology and pathway enrichment analysis.

**Fig 1:**
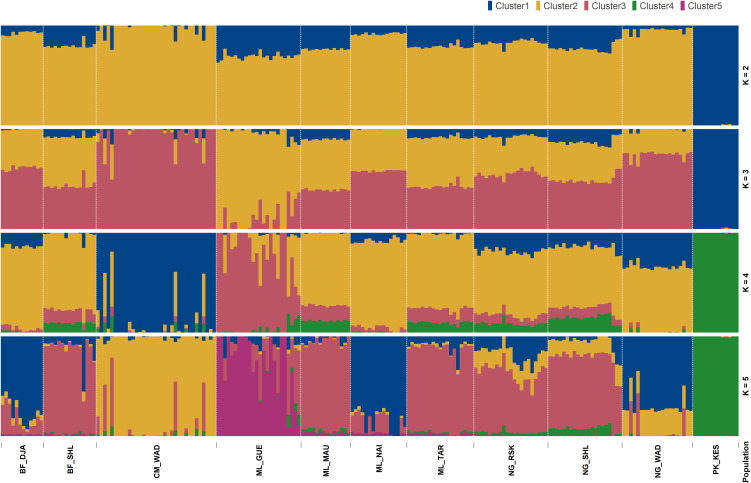
ADMIXTURE-inferred population structure of the West Africa–Cameroon transboundary livestock corridor and an outgroup. Each individual (vertical bar) is partitioned into coloured segments representing membership coefficients in K ancestral clusters estimated with ADMIXTURE for K = 2−5. Populations are ordered by country and population label: Djallonké (BF_DJA) and Sahel (BF_SHL) from Burkina Faso; West African Dwarf from Cameroon (CM_WAD); Guéra (ML_GUE), Maure (ML_MAU), Naine (ML_NAI) and Targui (ML_TAR) from Mali; Red Sokoto (NG_RSK), Sahel (NG_SHL) and West African Dwarf (NG_WAD) from Nigeria; and the Koh-e-Sulmani outgroup from Pakistan (PK_KES). Dashed white lines separate populations.

To assess whether genomic regions identified by DCMS were enriched for specific biological functions or pathways, we performed overrepresentation analyses of Gene Ontology (GO) and Kyoto Encyclopedia of Genes and Genomes (KEGG) terms using caprine gene symbols as the analysis space. We restricted the functional analyses to entries with a non-missing official gene symbol to avoid artefacts from poorly annotated loci. Genes overlapping DCMS outlier windows (q < 0.05) were assigned to four structure-informed genomic groupings defined from the population-structure analyses (see Fig 1) and geography: CM_WAD, the Djallonké/WAD genomic background (BF_DJA, ML_NAI, NG_WAD), the Sahel–Sudan genomic background (BF_SHL, ML_MAU, ML_TAR, NG_RSK, NG_SHL) and ML_GUE. For each grouping, we compared the resulting gene list against a background of all protein-coding *Capra hircus* genes with an annotated gene symbol in the ARS1 assembly in Ensembl, discarding loci without a recognised symbol. Enrichment for GO Biological Process (BP), Cellular Component (CC) and Molecular Function (MF) terms, and for KEGG pathways, was tested with *g:Profiler* (*gprofiler2* R interface; Kolberg et al. [54]) using *Capra hircus* (“chircus”) as the target organism and Benjamini–Hochberg FDR-adjusted p-values to account for multiple testing; terms with FDR ≤ 0.05 were considered significant. All significant GO and KEGG terms, together with their contributing genes, were retained. For groupings with enriched terms, term–gene relationships were summarised in bipartite term–gene network diagrams generated in R (*igraph* v 2.1.1 [55,56]).

## Results

### Population structure

ADMIXTURE cross-validation supported a model with five ancestry components. The cross-validation error declined from 0.657 at K = 1 to 0.639, 0.626 and 0.618 at K=2−4, reached a minimum of 0.614 at K = 5, and then increased to 0.6142 and 0.6252 at K = 6 and 7, respectively (S1 Fig). We therefore interpreted population structure at K = 5 (Fig 1; S1 Table).

At K = 5, the 209 unrelated goats formed five major ancestry clusters that broadly corresponded to geography and population labels (Fig 1; S1 Table). Cluster 1 predominated in the WAD populations: BF_DJA, ML_NAI and NG_WAD had mean (± standard error, SE) Cluster-1 assignments of 75.7 ± 3.6%, 83.4 ± 2.8% and 69.1 ± 2.7%, respectively, with only modest contributions from the other clusters. Cluster 2 was almost specific to the CM_WAD population, with an average of 89.7 ± 3.6% Cluster-2 ancestry and low contributions from the remaining clusters. A third component (Cluster 3) was characteristic of the Sahel–Sudan cluster. BF_SHL, ML_MAU, ML_TAR, NG_RSK and NG_SHL all showed Cluster-3 as their dominant ancestry, with mean proportions of 84.3 ± 4.0%, 89.2 ± 1.7%, 84.4 ± 1.4%, 60.0 ± 1.9% and 71.0 ± 1.8%, respectively (S1 Table). Within this group, NG_RSK and NG_SHL showed greater admixture, carrying larger contributions from Clusters 1 and 2 (e.g., Cluster-1 and Cluster-2 means of 17.9 ± 1.9% and 18.5 ± 0.6% in NG_RSK, and 6.0 ± 1.8% and 15.6 ± 0.6% in NG_SHL) than the Malian Sahelian populations. The ML_GUE breed harboured a distinct ancestry component (Cluster 5), with a mean Cluster-5 proportion of 73.6 ± 6.1% and only moderate Cluster-3 contribution (18.6 ± 4.4%). In contrast, the PK_KES was almost entirely assigned to Cluster 4, with 99.6 ± 0.2% of its genome in this component and negligible contributions from the other clusters (S1 Table). Cluster 4 occurred only at trace levels in the African corridor populations. In contrast, Cluster 5 was largely confined to ML_GUE and was present only at low levels in other corridor populations (Fig 1).

**Fig 2:**
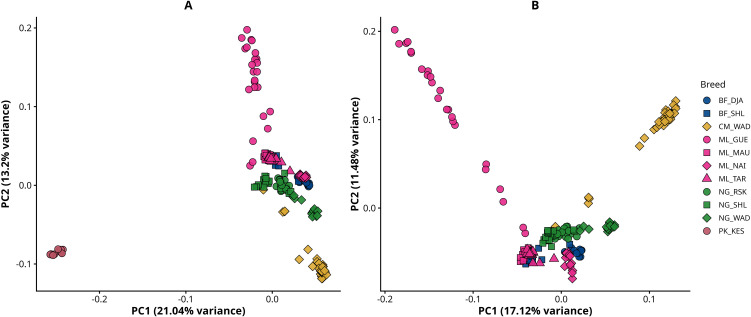
Principal component analysis of genome-wide SNP variation in the West Africa–Cameroon transboundary livestock corridor and an outgroup. Panel A includes the outgroup Koh-e-Sulmani (PK_KES); Panel B focuses on Nigerian (Red Sokoto – NG_RSK, Sahel – NG_SHL, West African Dwarf – NG_WAD) and neighbouring populations from Burkina Faso (Djallonké – BF_DJA, Sahel – BF_SHL), Mali (Maure – ML_MAU, Naine – ML_NAI, Guéra – ML_GUE, Targui – ML_TAR), and Cameroon (West African Dwarf – CM_WAD).

With all 11 populations included, principal component (PC) 1 (21.04% of variance) clearly separated the PK_KES from the African goats, which formed a compact cluster (Fig 2A). Within the African corridor panel, PC2 (13.2%) distinguished ML_GUE from the other populations. CM_WAD was shifted towards positive PC1 and negative PC2, and the remaining populations overlapped broadly. When the analysis was restricted to the 10 African corridor populations (excluding PK_KES), the separation among local genomic clusters became clearer (Fig 2B). PC1 (17.12%) captured a gradient with ML_GUE and CM_WAD at opposite ends, whereas the Djallonké–Naine–NG_WAD cluster and the Sahel–Sudan cluster populations clustered in intermediate, partially overlapping positions.

### Genetic differentiation

**Fig 3:**
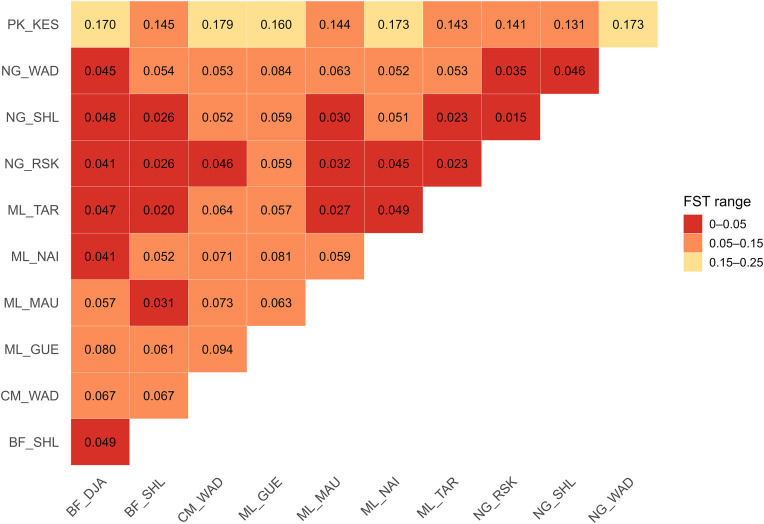
Pairwise Weir–Cockerham $F_{ST}$ among West Africa–Cameroon transboundary livestock corridor and an outgroup. Shown are pairwise $F_{ST}$ estimates for Nigerian (Red Sokoto – NG_RSK, Sahel – NG_SHL, West African Dwarf – NG_WAD), neighbouring populations from Burkina Faso, Mali, and Cameroon (Djallonké – BF_DJA, Sahel – BF_SHL, West African Dwarf – CM_WAD, Maure – ML_MAU, Naine – ML_NAI, Guéra – ML_GUE, Targui – ML_TAR) and outgroup (Koh-e-Sulmani – PK_KES) goat breeds.

Pairwise Weir–Cockerham $F_{ST}$ among the 11 sampled populations (55 contrasts; Fig 3) ranged from 0.015 to 0.179. Using bins of 0–0.050 (low), 0.050–0.150 (moderate) and 0.150–0.250 (high), there were 21 low, 29 moderate and 5 high comparisons. All within-country contrasts in Nigeria were low (NG_RSK–NG_SHL = 0.015, NG_WAD–NG_RSK = 0.035, NG_WAD–NG_SHL = 0.046). Differentiation within the Sahel-Sudan cluster (BF_SHL, ML_MAU, ML_TAR, NG_RSK, NG_SHL) was similarly low (0.015–0.032), whereas the Djallonké/WAD cluster (BF_DJA, ML_NAI, NG_WAD) showed slightly higher but still low-to-borderline-moderate values (0.041–0.052). ML_GUE was moderately differentiated from the other corridor populations (0.057–0.094), and CM_WAD also showed mostly moderate $F_{ST}$ relative to the remaining populations (0.046–0.094). All corridor breeds were moderately to highly differentiated from the outgroup PK_KES (0.131–0.179), with the largest value between PK_KES and CM_WAD (0.179). Overall, differentiation was lowest within the structure-defined clusters and highest against the outgroup, with elevated values also involving ML_GUE and CM_WAD.

### Historical gene flow

**Fig 4:**
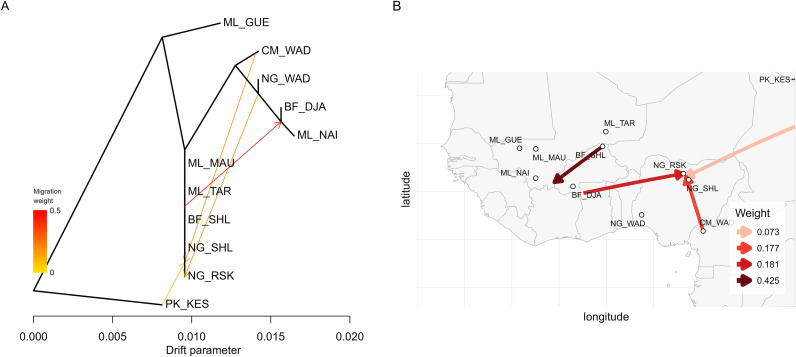
Population relationships and inferred gene flow among West Africa–Cameroon transboundary livestock corridor and an outgroup. (A) TreeMix maximum-likelihood graph (m = 4) showing four migration edges. (B) Cartographic rendering of the same four edges over West Africa and into Cameroon; arrow colour encodes edge weight. Breeds: NG_RSK (Red Sokoto), NG_SHL (Sahel), NG_WAD (West African Dwarf); BF_DJA (Djallonké), BF_SHL (Sahel), CM_WAD (West African Dwarf), ML_MAU (Maure), ML_NAI (Naine), ML_GUE (Guéra), ML_TAR (Targui); outgroup PK_KES (Koh-e-Sulmani).

TreeMix graphs were fitted with 0–6 migration edges. We retained m = 4 for interpretation based on likelihood and residual-covariance diagnostics (Fig 4, S1 File). In the maximum-likelihood tree (Fig 4A), PK_KES forms the outgroup, with all African breeds on a single derived branch. Within West Africa–Cameroon, ML_GUE splits earliest, whereas CM_WAD, NG_WAD, BF_DJA and ML_NAI cluster together and the Sahel-Sudan cluster populations (BF_SHL, ML_MAU, ML_TAR, NG_RSK, NG_SHL) form a second subtree, consistent with the PCA and ADMIXTURE groupings. Four migration edges are inferred: a low-weight edge from PK_KES to NG_RSK (weight 0.073), two intermediate edges from NG_RSK to BF_DJA (0.177) and from NG_SHL to CM_WAD (0.181), and a strong edge from ML_MAU to BF_SHL (0.425). Given the outgroup placement and the potential influence of SNP-chip ascertainment, we did not interpret the PK_KES to NG_RSK edge as evidence of recent gene flow. The cartographic rendering (Fig 4B) shows these edges along an east–west axis across the Sahel and into neighbouring WAD/Djallonké populations, connecting Nigerian, Malian and Burkinabè goats.

### Timing of admixture events

**Fig 5:**
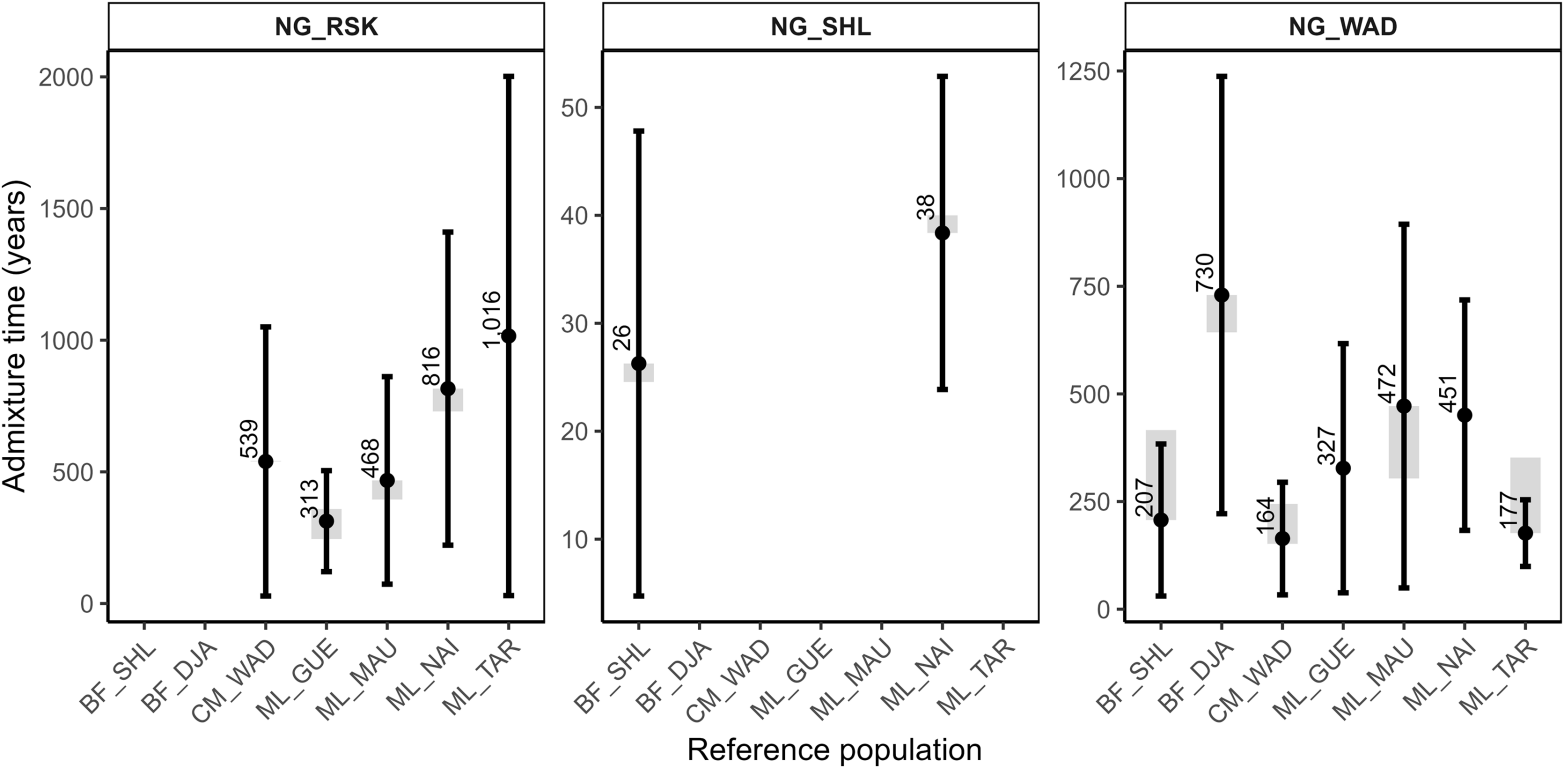
ALDER-based timing of admixture episodes involving Nigerian goat breeds across the West Africa–Cameroon transboundary livestock corridor. Admixture times (years, assuming a 3.7-year generation interval) for Nigerian goat breed populations NG_RSK, NG_SHL and NG_WAD with corridor reference populations. Black points and error bars show the best estimate and ± standard error at the LD start distance with the highest Z-score; grey bands indicate the range of dates across start distances (0.3–0.7 cM).

ALDER identified 14 target–reference pairs with significant one-reference LD decay (Z ≥ 2.0 at ≥1 LD start distance; Fig 5; S2 Table). Seven target reference combinations had no significant LD-decay fit across the evaluated start-distance grid and are reported in S2 Table but not reflected in Fig 5. Across the significant pairs, sensitivity to the LD start distance varied: several pairs remained significant across multiple adjacent thresholds (classified as stable/consistent in S2 Table), whereas others were supported at only a single threshold and are therefore treated as fragile. Among the 14 significant pairs, four were classified as stable, six as consistent, and four as fragile. For NG_RSK, one-reference fits place admixture-related LD on centennial to millennial scales, with point estimates of about 313 years (ML_GUE), 468 years (ML_MAU), 539 years (CM_WAD), 816 years (ML_NAI), and a more weakly supported older estimate of 1,016 years (ML_TAR). The ML_TAR estimate was classified as fragile and is therefore interpreted cautiously. NG_SHL shows very recent admixture-related LD with BF_SHL (26 years) and ML_NAI (38 years), both classified as stable and consistent with recent livestock exchange among Sahelian and neighbouring corridor populations. NG_WAD shows multiple centennial-scale signals spanning 160–730 years across several references (Fig 5), with uncertainty increasing for older dates. For NG_WAD, CM_WAD was classified as stable, BF_SHL, ML_MAU and ML_TAR as consistent, and BF_DJA, ML_GUE and ML_NAI as fragile. Grey bands summarise start-distance sensitivity across $d_{min}=\text{0}.\text{3}-\text{0}.\text{7}$ cM. Full outputs and stability classifications are provided in S2 Table. These dates should be interpreted as LD-decay proxy dates rather than exact historical events.

### Demographic history

**Fig 6:**
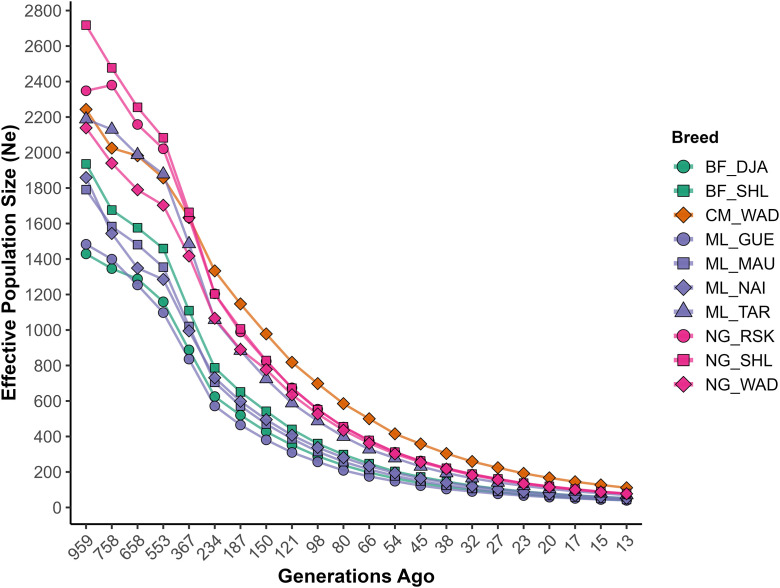
Historical trends in effective population size across the West Africa–Cameroon transboundary livestock corridor. LD-based effective population size ($N_{e}$) trajectories reconstructed with SNeP for Nigerian (NG_RSK, NG_SHL, NG_WAD), neighbouring corridor populations (BF_DJA, BF_SHL, CM_WAD, ML_GUE, ML_MAU, ML_NAI, ML_TAR), plotted over 22 generational time points from 13 to 959 generations ago.

$N_{e}$ was reconstructed at 22 time points from 13 to 959 generations ago (Fig 6). At 959 generations, $N_{e}$ ranged from 1,429 (BF_DJA) to 2,718 (NG_SHL); other large values were 2,243 (CM_WAD), 2,188 (ML_TAR), 2,139 (NG_WAD) and 2,348 (NG_RSK), with ML_GUE among the lowest at 1,483. By 367 generations ago, $N_{e}$ had declined in all breeds to 836–1,667, with ML_GUE at 836 and NG_SHL at 1,663 (CM_WAD and NG_RSK both at 1,633; NG_WAD at 1,417). At 150 generations ago, the range narrowed further to 381–978 (ML_GUE 381, CM_WAD 978, NG_SHL 828, NG_RSK 824). At the most recent point (13 generations ago), all breeds converged below 120: BF_DJA 42, BF_SHL 52, CM_WAD 111, ML_GUE 40, ML_MAU 45, ML_NAI 52, ML_TAR 70, NG_RSK 77, NG_SHL 79 and NG_WAD 77. Overall, $N_{e}$ decreased monotonically through time in every breed, with NG_SHL and NG_RSK remaining among the largest and ML_GUE consistently the smallest.

ROH-based genomic inbreeding varied among the African corridor populations but was generally low to moderate (S6 Table). Mean $F_{ROH}$ ranged from 0.004 in ML_MAU to 0.040 in ML_GUE. The highest values were observed in ML_GUE, NG_RSK, CM_WAD, ML_NAI and NG_WAD, whereas ML_MAU showed the lowest ROH burden. In ML_GUE and NG_RSK, a substantial proportion of $F_{ROH}$ was contributed by long ROH segments (>8 Mb), consistent with greater recent autozygosity in these populations. Overall, the ROH results indicate population-specific differences in recent inbreeding despite broadly low genome-wide ROH levels across the corridor.

### Genome-wide signatures of selection (DCMS)

**Fig 7:**
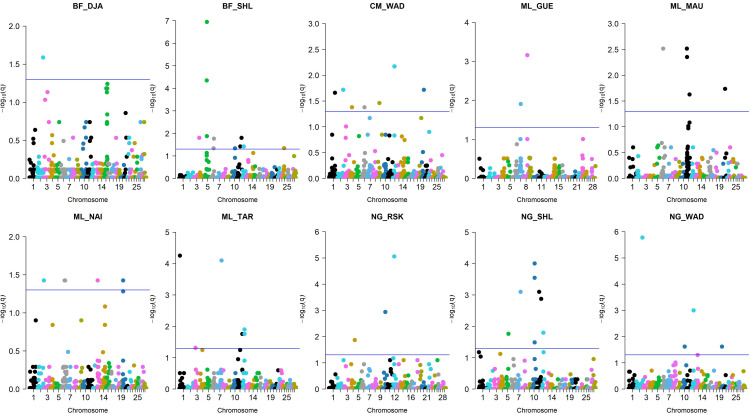
Genome-wide DCMS selection scan across the West Africa–Cameroon transboundary livestock corridor. Breed-wise DCMS values for fixed 500-kb windows across the autosomes. Each point represents a window coloured by chromosome; the horizontal line marks the genome-wide significance threshold (q ≤ 0.05). Breed codes: BF_DJA, BF_SHL, CM_WAD, ML_GUE, ML_MAU, ML_NAI, ML_TAR, NG_RSK, NG_SHL, NG_WAD.

Breed-wise DCMS scans were carried out on fixed 500-kb windows, with significance defined at q ≤ 0.05 (Fig 7). Across the ten breeds, 53 windows surpassed this threshold (S3 Table). Per-breed counts were: BF_DJA = 1, BF_SHL = 11, CM_WAD = 8, ML_GUE = 2, ML_MAU = 5, ML_NAI = 5, ML_TAR = 6, NG_RSK = 3, NG_SHL = 8, and NG_WAD = 4. The most extreme DCMS values were observed in BF_SHL (max − log₁₀q ≈ 6.95), NG_WAD (≈ 5.78), NG_RSK (≈ 5.06) and ML_TAR (≈ 4.25), whereas BF_DJA and ML_NAI showed only modest elevations just above the significance threshold. Significant windows were distributed over 1–7 chromosomes per breed, with BF_SHL and CM_WAD showing the broadest chromosomal coverage.

Several windows were shared among breeds (S3 Table). A window on chromosome 2 (118.5–119.0 Mb) was significant in all three Djallonké/WAD cluster populations (BF_DJA, ML_NAI, NG_WAD). Additional shared regions occurred on chromosome 6 (57.0–57.5 Mb; CM_WAD, ML_MAU, ML_NAI), chromosome 7 (87.0–87.5 Mb; ML_TAR, NG_SHL), chromosome 10 (59.5–60.0 Mb; BF_SHL, NG_RSK, NG_SHL; 60.0–60.5 Mb; NG_SHL, NG_WAD), chromosome 11 (68.5–69.0 Mb; BF_SHL, ML_MAU; 93.0–93.5 Mb; BF_SHL, ML_TAR, NG_SHL) and chromosome 12 (34.5–35.0 Mb; BF_SHL, ML_TAR, NG_SHL). The most widely shared signal was the chromosome-12 window at 35.5–36.0 Mb, which was significant in CM_WAD, ML_TAR, NG_RSK, and NG_WAD, thus spanning the CM_WAD lineage, the Sahel-Sudan cluster, and the Djallonké/WAD cluster. These patterns indicate both background-aligned signals, such as the chromosome-2 Djallonké/WAD window and Sahel–Sudan shared windows on chromosomes 10–12, and cross-background windows that are better treated as corridor-level candidates rather than background-specific signals.

Annotation of the 53 DCMS windows yielded 202 genes with assigned symbols and 83 additional features without gene symbols (S4 Table). Within the Nigerian breeds, the number of genes with symbols was 15 in NG_RSK, 37 in NG_SHL and 23 in NG_WAD. Complete genomic coordinates, DCMS statistics and per-window gene content are provided in S3 and S4 Tables.

### Functional annotation and enrichment of candidate regions

Genes within DCMS outlier windows were analysed separately for each structure-informed genomic group. Across all groups, 52 terms passed the enrichment threshold (FDR ≤ 0.05), comprising 32 GO CC, 10 BP, 9 MF terms and 1 KEGG pathway (S5 Table). No significant GO or KEGG term was detected for the CM_WAD population.

**Fig 8:**
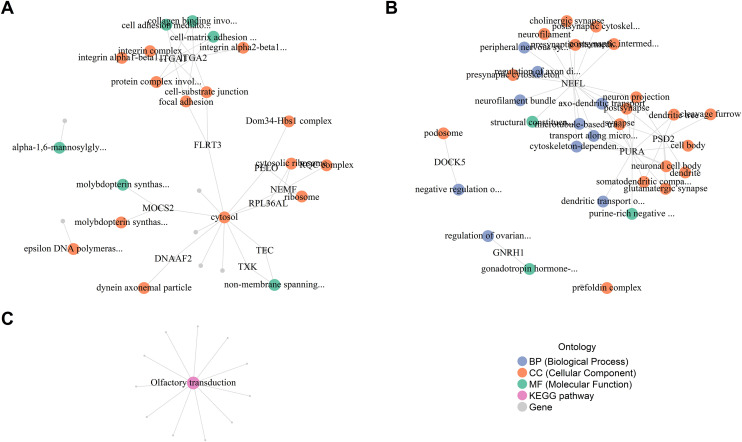
Group-specific GO/KEGG term–gene networks from DCMS candidate regions in goat populations from the West Africa–Cameroon transboundary livestock corridor. Panel A: Djallonké–Naine–NG_WAD cluster; Panel B: Guéra; Panel C: Sahel–Sudan cluster. Nodes represent enriched GO (BP, CC, MF) or KEGG terms (coloured) and their member genes (grey); edges connect genes to the terms in which they occur. Only terms with FDR ≤ 0.05 are shown; CM_WAD yielded no significant terms.

For the Djallonké/WAD group, 20 terms were enriched (14 CC, 6 MF). CC terms comprised mainly cytosolic and adhesion-related components, including cytosolic ribosomes, cytosol, focal adhesions, integrin complexes, cell-substrate junctions, and protein complexes involved in cell adhesion. MF terms highlighted collagen binding involved in cell–matrix adhesion, cell-matrix adhesion mediator activity, cell adhesion mediator activity, non-membrane spanning protein tyrosine kinase activity and molybdopterin synthase activity. Sixteen genes underpinned these terms; *ITGA1* and *ITGA2* occurred in the largest number of enriched terms (8 each), linking the integrin and focal-adhesion nodes, while *NEMF*, *PELO* and *RPL36AL* jointly connected the cytosolic ribosome, ribosome and RQC-complex terms (Fig 8A; S5 Table).

For ML_GUE, 31 terms were enriched (10 BP, 18 CC, 3 MF), yielding a set of enriched terms largely associated with neuronal and synaptic structures (Fig 8B). BP terms centred on axonal and dendritic transport and microtubule-based trafficking (e.g., axo-dendritic transport, transport along microtubule, cytoskeleton-dependent intracellular transport, neurofilament bundle assembly), together with negative regulation of vascular-associated smooth muscle contraction and regulation of ovarian follicle development. CC terms were mainly mapped to synaptic and neuronal structures, including postsynapse, neurofilament, neuron projection, dendrite, dendritic tree, cholinergic synapse, and glutamatergic synapse. MF terms involved gonadotropin hormone-releasing hormone activity, purine-rich negative regulatory element binding and structural constituent of postsynaptic intermediate filament cytoskeleton. Six genes contributed to these terms; *NEFL*, *PURA* and *PSD2* occurred most frequently among the enriched terms (17, 15 and 10 terms, respectively), with additional contributions from *DOCK5*, *GNRH1* and *PFDN1* (S5 Table).

In the Sahel-Sudan cluster, enrichment was restricted to a single KEGG pathway, Olfactory transduction (KEGG:04740; Fig 8C). This term was supported by 11 olfactory receptor genes (*OR10R2*, *OR10Z1*, *OR1J1*, *OR1J2*, *OR1L1*, *OR1Q1*, *OR5C1*, *OR6K2*, *OR6K6*, *OR6N1*, *OR6P1*), each contributing once to the pathway (S5 Table).

## Discussion

This transboundary reanalysis asked whether widely reused vernacular breed labels across West Africa map to coherent genomic backgrounds, while also reconstructing connectivity through time, characterising demography and inbreeding, and prioritising candidate adaptive regions in a structure-aware framework. Our integrated results place NG_SHL, NG_RSK, and NG_WAD within a West Africa–Cameroon genomic context that is better represented by shared regional backgrounds than by country-bounded “breeds,” consistent with the eco-geographic structuring and admixture emphasised in AdaptMap [2]. Across complementary analyses (structure, differentiation, admixture dating, demography, and multi-statistic selection scans), three major backgrounds are supported: a Sahel–Sudan background spanning BF_SHL–ML_MAU–ML_TAR–NG_RSK–NG_SHL, a Djallonké/WAD background spanning BF_DJA–ML_NAI–NG_WAD, and a distinct Cameroon dwarf lineage represented by CM_WAD, with ML_GUE occupying a more drifted position within the Sahelian continuum.

### Genomic structure and the meaning of recurrent breed labels across borders

A practical premise in livestock systems is that widely reused labels (e.g., “Sahel,” “West African Dwarf/Djallonké”) denote discrete biological units that can be monitored and improved. West African goat systems, however, are shaped by exchange, especially trade and seasonal mobility, so these labels may also track ecotype-linked phenotypes that persist despite recurrent movement and admixture, rather than stable genomic units. Early microsatellite work already pointed to high within-population diversity [16] and generally modest differentiation shaped by geography and exchange [17]. What the present analysis adds is an explicit cross-border test of when vernacular labels align with shared ancestry and when a single name spans multiple genomic entities.

Within the Sahel–Sudan background (BF_SHL, ML_MAU, ML_TAR, NG_RSK, NG_SHL), the shared dominant ancestry component (Cluster 3), tight clustering in PCA, and low within-group $F_{ST}$ jointly support interpretation as one transboundary genomic background rather than separate national Sahel “breeds.” This aligns with evidence that Sahelian populations are only weakly structured at the regional scale where exchange is common [17]. ML_GUE deviates from this pattern by exhibiting a distinct ADMIXTURE component (Cluster 5; Fig 1) and higher differentiation (Fig 3), consistent with drift-amplified divergence within a Sahelian/Sudanian continuum rather than an entirely separate ecotype.

For dwarf/Djallonké types, NG_WAD, ML_NAI, and BF_DJA form a coherent Djallonké/WAD background, sharing a dominant ancestry component (Cluster 1; Fig 1) and overlapping PCA positions (Fig 2B), with low-to-borderline-moderate differentiation among them (Fig 3). This accords with the long-standing view that Djallonké-type populations can remain weakly differentiated across space under exchange [16]. CM_WAD, by contrast, is near-fixed for a distinct ancestry component (Cluster 2; Fig 1) and sits apart on the PCA gradient (Fig 2B), suggesting deeper divergence and/or more episodic connectivity across the Nigeria–Cameroon interface than within the Sahel belt. Genome-wide analyses have previously flagged Cameroon goats as differentiated within broader African panels [3]. The practical implication is straightforward: the label “West African Dwarf” does not represent a single genomic unit here; it spans at least a Djallonké/WAD background (NG_WAD–ML_NAI–BF_DJA) and a distinct Cameroon dwarf lineage (CM_WAD).

NG_RSK is often treated as exceptional in production narratives, including for its association with valued skins/leather in West African systems [57]. Genomically, however, it is embedded within Sahel–Sudan, as NG_RSK shares the dominant Sahel–Sudan component (Fig 1), shows extremely low $F_{ST}$ to NG_SHL (Fig 3), and clusters with Sahelian populations in PCA/TreeMix (Figs 2–4). The same close relationship between Maradi/Red Sokoto and Sahel goats relative to WAD was also reported in Nigerian microsatellite data [58]. Taken together, Red Sokoto is best interpreted as a phenotypically salient node within a broader Sahel–Sudan genomic background rather than as a deeply subdivided lineage.

### Connectivity has depth: decadal exchange layered onto older admixture

Connectivity in this corridor is time-layered rather than episodic. ALDER estimates admixture time from the exponential decay of weighted linkage disequilibrium with genetic distance [30]. In NG_SHL, significant one-reference fits using BF_SHL and ML_NAI as proxies place admixture-linked LD at decadal time scales under the assumed generation interval (Fig 5; S2 Table). Because one-reference curves can be sensitive to proxy choice and to residual/background LD [30], these estimates are best read as evidence of very recent or ongoing corridor connectivity rather than as a precise historical timestamp, consistent with shallow differentiation within Sahel–Sudan (Fig 3) and the dominant Sahel–Sudan ancestry component spanning multiple countries (Fig 1). Older layers appear in NG_RSK and NG_WAD, where ALDER signals extend to centuries-scale time depths and involve both Sahelian and dwarf references (Fig 5; S2 Table). These deeper signals are consistent with sustained exchange across Sahel–savanna–forest interfaces, as emphasised in accounts of West African livelihood strategies and landscape-linked mobility [8] and in historical analyses of markets, governance, and rural change that structure exchange corridors over long periods [9]. At the genetic level, this is compatible with microsatellite evidence for structure and Sahelian introgression into Djallonké-type goats [17].

TreeMix provides an independent, allele-frequency-based summary of drift and migration by fitting a maximum-likelihood graph with migration edges [29]. The inferred edges linking Sahel–Sudan nodes and connecting Sahelian and dwarf populations reinforce a system shaped by repeated exchange rather than isolated introgression. Inference should remain bounded, because TreeMix is a simplified model of complex demographic histories and edge direction/weight can depend on sampling and model specification [29]. Nonetheless, concordance across ADMIXTURE/PCA (Figs 1, 2), $F_{ST}$ (Fig 3), TreeMix (Fig 4), and ALDER (Fig 5) supports a central conclusion: transboundary mobility is a persistent feature of the system, with very recent connectivity superimposed on older Sahel–dwarf exchange. Contemporary syntheses of West African transhumance similarly emphasise that seasonal mobility and trade remain major forces structuring livestock contact networks and cross-border circulation [10].

### Demographic contraction and ROH-based inbreeding: connectivity does not preclude local drift

Despite evidence of connectivity, LD-based $N_{e}$ trajectories indicate sustained decline across ecotypes, converging to low values in the most recent interval (Fig 6). LD-based $N_{e}$ inference from SNP data has a clear theoretical basis [34] and is widely used for recent demographic reconstruction in livestock [31]. Comparable genomic evidence consistent with demographic contraction and reduced effective population size has been reported in other goat populations [59,60]. From a conservation-genetic perspective, small contemporary effective size increases the expected rate of drift and the risk of losing adaptive variation if contraction persists [61].

ROH profiles complement $N_{e}$ by estimating autozygosity within individuals and separating older from more recent inbreeding through ROH length distributions [38]. Practical interpretation anchors for ROH segment lengths as indicators of recent versus older shared ancestry are also well established [39]. In this dataset, mean $F_{ROH}$ varies among populations, but long ROH, consistent with more recent common ancestry, contribute notably in ML_GUE and NG_RSK. This pattern is consistent with locally narrow breeding pools, even when the wider system remains connected.

ROH signals should not be treated as a direct proxy for specific management decisions in the absence of metadata (e.g., pedigree/sire use, herd size, movement), because the same ROH profile can arise from recent consanguinity or persistent small $N_{e}$ [38], and because segment distributions are shaped by both demography and recombination history [39]. What ROH can support more directly is a “where to look” inference: long segments indicate recent shared ancestry within local mating pools, regardless of whether it reflects deliberate close-kin mating or chronic restriction of breeders. In that context, the coexistence of cross-border connectivity and population-specific long ROH argues against assuming that exchange alone will uniformly buffer inbreeding risk. Practically, this supports explicit inbreeding management at herd/community scale, including avoiding indiscriminate mating, avoiding repeated matings among close relatives, limiting over-reliance on a small number of related males, and periodically introducing unrelated breeding males even where cross-border exchange is common.

### Ecotype-specific polygenic selection and functional themes: pathway-level differentiation in a corridor frame

DCMS combines multiple selection statistics to increase robustness and power relative to single-scan approaches, while explicitly accounting for redundancy among statistics [50]. Here, we used empirical p-values with correlation-aware weighting, a standard approach in multi-statistic scans [49]. The modest number of DCMS-significant windows across breeds (S3 Table), together with enrichment that is strongest at the pathway/term level (S5 Table), is consistent with adaptation dominated by polygenic and/or soft-sweep architectures rather than classic hard sweeps [47]. Accordingly, we use enrichment to organise and prioritise recurring functional signals across many windows, rather than to argue for single “major genes.” We therefore interpret the DCMS results at two levels: shared or background-aligned genomic windows, and functional themes that generate phenotype-facing hypotheses for each genomic background. Selection-signature studies in African goats commonly add GO/KEGG enrichment to interpret candidate regions and summarise functional categories [20,62].

**Djallonké/WAD background (BF_DJA–ML_NAI–NG_WAD):** Enrichment for focal adhesion and integrin-complex terms (Fig 8A; S5 Table) highlights cell–matrix interaction pathways. Integrins sit at the centre of bidirectional signalling between cells and the extracellular matrix [63], making this theme mechanistically plausible in relation to tissue integrity and remodelling demands under chronic challenge. The prominence of *ITGA1*/*ITGA2* in the enriched term–gene network (Fig 8A; S5 Table) is consistent with this interpretation. Integrins can also act as entry or interaction points for diverse pathogens at epithelial and endothelial interfaces [64], so “adhesion biology” is a plausible arena for host–environment negotiation even when causality cannot be assigned to individual genes from SNP-chip scans alone. Phenotypically, this points most naturally to interfaces that take repeated damage in humid and sub-humid systems, especially gut and skin: barrier integrity, wound repair, inflammatory cell trafficking, and tolerance to repeated parasite exposure are all ecotype-relevant axes. The co-enrichment of ribosome-associated quality-control components in this group points to translational stress management as an additional axis of differentiation. WAD goats have a long empirical record of helminth resilience and trypanotolerance [13–15,65], and the enrichment profile is compatible with barrier/adhesion and cellular stress-response modules contributing to local fitness. In practical terms, it narrows follow-up to testable mechanisms (e.g., parasite burden, anaemia/PCV dynamics, growth under challenge, skin lesion scores, and immune readouts) rather than repeating broad “candidate gene” narratives.

**ML_GUE (drifted Sahelian subgroup):** ML_GUE shows an enrichment profile dominated by neuronal and synaptic terms (Fig 8B; S5 Table). Several highlighted loci have established roles in neurobiology and neuroendocrine systems. *NEFL*, for instance, is central to neurofilament structure and axonal physiology [66] and is widely used as a marker of neuro-axonal integrity [67]. In this dataset, the concentration of signal in transport/synapse-associated terms is consistent with selection acting on neuroendocrine and behavioural pathways that influence stress responsiveness, movement ecology, or reproductive timing. That is ecologically plausible in Sahel-linked production systems where heat load, dehydration risk, long-distance movement, and seasonal feed gaps can strongly shape behaviour, grazing persistence, and reproductive scheduling, even if the present dataset cannot map any one locus to any one phenotype. Two considerations nonetheless bound inference: ML_GUE is differentiated and demographically constrained, and drift can inflate sweep-like signals in such populations. The value of this synthesis is therefore prioritisation with restraint: ML_GUE candidates are targets for validation, not definitive “adaptive genes.”

**Sahel–Sudan background:** In Sahel–Sudan, enrichment is restricted to “olfactory transduction,” concentrating signal on olfactory receptor (*OR*) loci (Fig 8C; S5 Table). *OR* families evolve rapidly and often appear in selection scans because they comprise large, dynamic gene repertoires [68]. We therefore treat this enrichment as a testable hypothesis rather than a phenotype claim: selection in Sahelian production contexts may have acted on chemosensory sensitivity, with potential downstream effects on forage discrimination and/or social/pheromonal cue perception under extensive grazing and mobility. A competing explanation is that the signal reflects gene-family architecture and tagging/ascertainment effects rather than selection on a specific behavioural trait. Notably, *OR*/chemosensory loci have also appeared in goat selection-scan studies framed around environmental adaptation [20,69], but discriminating biology from architecture will require higher-resolution follow-up (e.g., whole-genome sequencing to resolve copy-number/structural variation) and matched phenotypes.

Across groups, annotation remains a practical constraint. A substantial fraction of candidate features lacks official gene symbols (S4 Table), reflecting incomplete caprine annotation [70] and the prevalence of lineage-specific loci and difficult-to-annotate gene families [71]. Because enrichment depends on named genes, pathway inference will undercount functional diversity in symbol-poor regions and may differentially affect groups with *OR*-rich windows. Operationally, the safest output of this section is a ranked, phenotype-facing hypothesis set: do these pathways track measurable ecotype-relevant outcomes (parasite burden, survival, body condition, reproductive timing, and heat-stress proxies) in the same populations?

The corridor frame makes the trade-off clear: cross-border exchange exists, but it does not automatically prevent local drift, so resilience breeding needs ecotype-relevant phenotyping plus routine inbreeding control at the community herd level.

### Methodological limitations and next steps

This study leverages CaprineSNP50 density to support LD-based demography, ROH profiling, and multi-statistic selection scanning in a transboundary sampling frame, but SNP arrays are subject to ascertainment bias [2,72]. Such bias can affect allele-frequency distributions, population differentiation and linkage-disequilibrium patterns, with consequences for both selection scans and LD-based demographic inference [72]. For selection scans, this may reduce power to detect population-specific or poorly tagged variants and may shift inference toward regions well represented on the array [72]. LD-based $N_{e}$ estimates should therefore be interpreted comparatively rather than as precise census correlates [31,34]. Admixture dates from ALDER should likewise be read as the timing of admixture-linked LD decay rather than as direct dates of discrete historical events [30]. Although DCMS reduces reliance on any single selection statistic by combining complementary signals, modest sample sizes in some populations can limit the strength of population-specific inference. We therefore treat DCMS windows as prioritised candidate regions for replication and phenotype-based validation rather than as confirmed adaptive loci.

A direct way to strengthen inference is an explicitly transboundary sequencing and phenotyping program: whole-genome sequencing across the inferred backgrounds; denser sampling of under-represented corridor nodes; and matched phenotypes/management metadata. Such data would allow fine-mapping of DCMS windows, explicit modelling of structural variation (especially in *OR*-rich regions), and formal tests of genotype–environment associations that translate these structure-defined genomic backgrounds into operational conservation units and improvement zones. This is also the scale at which breeding and conservation frameworks that emphasise locally appropriate designs and sustainable diversity management can be implemented.

## Conclusions

By jointly analysing genomic structure, admixture chronology, LD-based $N_{e}$ trajectories, ROH, and pathway-level selection signals, this study moves beyond country-bounded “breeds” to corridor-scale genomic backgrounds across the West Africa–Cameroon system. Three backgrounds are supported: Sahel–Sudan (Burkina Faso–Mali–Nigeria), Djallonké/WAD (Burkina Faso–Mali–Nigeria), and a distinct Cameroon dwarf lineage, with Guéra a drifted Sahelian subgroup. Recent exchange is evident, but declining $N_{e}$ and population-specific long ROH show that connectivity does not prevent local drift or recent autozygosity. DCMS prioritises testable functional themes (adhesion/translation quality control; neuronal/neuroendocrine; olfactory transduction) without implying causation, and the results support explicit local inbreeding management (avoid close-kin matings and over-use of related males) alongside sequencing and phenotype-enabled validation.

## Supporting information

S1 Fig Cross-validation profile for ADMIXTURE clustering of West Africa–Cameroon transboundary livestock corridor. (TIFF)

S1 Table Genome-wide ancestry proportions for the five genetic clusters inferred in ADMIXTURE analysis of goat populations from the West Africa–Cameroon transboundary livestock corridor and the PK_KES outgroup. (XLSX)

S2 Table ALDER start-distance sensitivity (Nigerian target populations vs corridor reference populations). (XLSX)

S3 Table DCMS-significant windows across Nigerian and neighbouring corridor populations. (XLSX)

S4 Table List of candidate genes identified in selection-signature regions across Nigerian breeds and neighbouring corridor populations. (XLSX)

S5 Table GO and KEGG terms enriched among genes in DCMS candidate regions of goat populations from the West Africa–Cameroon transboundary livestock corridor. (XLSX)

S6 Table Runs of homozygosity and genomic inbreeding in goat populations from the West Africa–Cameroon transboundary livestock corridor. (XLSX)

S1 File TreeMix graphs and residual covariance heatmaps for goat populations from the West Africa–Cameroon transboundary livestock corridor using PK_KES as outgroup. (PDF)

## References

[1] Zheng Z, Wang X, Li M, Li Y, Yang Z, Wang X, et al. The origin of domestication genes in goats. Sci Adv. 2020;6(21):eaaz5216. doi: 10.1126/sciadv.aaz5216 32671210 PMC7314551

[2] Colli L, Milanesi M, Talenti A, Bertolini F, Chen M, Crisà A, et al. Genome-wide SNP profiling of worldwide goat populations reveals strong partitioning of diversity and highlights post-domestication migration routes. Genet Sel Evol. 2018;50(1):58. doi: 10.1186/s12711-018-0422-x 30449284 PMC6240949

[3] Tarekegn GM, Wouobeng P, Jaures KS, Mrode R, Edea Z, Liu B, et al. Genome-wide diversity and demographic dynamics of Cameroon goats and their divergence from east African, north African, and Asian conspecifics. PLoS One. 2019;14(4):e0214843. doi: 10.1371/journal.pone.0214843 31002664 PMC6474588

[4] Galiè A, Teufel N, Korir L, Baltenweck I, Webb Girard A, Dominguez-Salas P, et al. The women’s empowerment in livestock index. Soc Indic Res. 2019;142: 799–825. doi: 10.1007/s11205-018-1934-z

[5] Cooke AS, Machekano H, Ventura-Cordero J, Louro-Lopez A, Joseph V, Gwiriri LC, et al. Opportunities to improve goat production and food security in Botswana through forage nutrition and the use of supplemental feeds. Food Secur. 2024;16(3):607–22. doi: 10.1007/s12571-024-01452-1 38770158 PMC11102351

[6] Wurzinger M, Sölkner J, Iñiguez L. Important aspects and limitations in considering community-based breeding programs for low-input smallholder livestock systems. Small Rumin Res. 2011;98(1–3):170–5. doi: 10.1016/j.smallrumres.2011.03.035

[7] Boettcher PJ, Hoffmann I, Baumung R, Drucker AG, McManus C, Berg P, et al. Genetic resources and genomics for adaptation of livestock to climate change. Front Genet. 2015;5:461. doi: 10.3389/fgene.2014.00461 25646122 PMC4298221

[8] Mortimore M. Adapting to drought: farmers, famines and desertification in West Africa. Cambridge: Cambridge University Press; 1989. doi: 10.1017/CBO9780511720772

[9] Fenske J. Ecology, trade, and states in pre-colonial Africa. J Eur Econ Assoc. 2014;12: 612–40.

[10] Timpong-Jones EC, Samuels I, Sarkwa FO, Oppong-Anane K, Majekodumni AO. Transhumance pastoralism in West Africa – its importance, policies and challenges. African J Range Forage Sci. 2023;40(1):114–28. doi: 10.2989/10220119.2022.2160012

[11] Yakubu A, Salako A, Imumorin I, Ige A, Akinyemi M. Discriminant analysis of morphometric differentiation in the West African Dwarf and Red Sokoto goats. S Afr J Anim Sci. 2010;40: 381–7. doi: 10.4314/sajas.v40i4.65261

[12] Wheto M, Ilori BM, Sanda AJ, Adeleke MA, Durosaro SO, Adenaike AS, et al. Morphological characterization and evaluation of heat tolerance traits in Nigerian goat breeds. Niger J Anim Prod. 2015;42: 1–13. doi: 10.51791/njap.v42i2.2613

[13] Behnke JM, Chiejina SN, Musongong GA, Nnadi PA, Ngongeh LA, Abonyi FO, et al. Resistance and resilience of traditionally managed West African Dwarf goats from the savanna zone of northern Nigeria to naturally acquired trypanosome and gastrointestinal nematode infections. J Helminthol. 2011;85(1):80–91. doi: 10.1017/S0022149X10000295 20459880

[14] Chiejina SN, Behnke JM. The unique resistance and resilience of the Nigerian West African Dwarf goat to gastrointestinal nematode infections. Parasit Vectors. 2011;4:12. doi: 10.1186/1756-3305-4-12 21291550 PMC3042002

[15] Chiejina SN, Behnke JM, Fakae BB. Haemonchotolerance in West African Dwarf goats: contribution to sustainable, anthelmintics-free helminth control in traditionally managed Nigerian dwarf goats. Parasite. 2015;22:7. doi: 10.1051/parasite/2015006 25744655 PMC4321401

[16] Missohou A, Poutya MR, Nenonene A, Dayo G-K, Ayssiwede SB, Talaki E, et al. Genetic diversity and differentiation in nine West African local goat breeds assessed via microsatellite polymorphism. Small Rumin Res. 2011;99(1):20–4. doi: 10.1016/j.smallrumres.2011.04.005

[17] Traoré A, Álvarez I, Fernández I, Pérez-Pardal L, Kaboré A, Ouédraogo-Sanou GM, et al. Ascertaining gene flow patterns in livestock populations of developing countries: a case study in Burkina Faso goat. BMC Genet. 2012;13:35. doi: 10.1186/1471-2156-13-35 22564289 PMC3413537

[18] Nandolo W, Mészáros G, Banda LJ, Gondwe TN, Lamuno D, Mulindwa HA, et al. Timing and extent of inbreeding in African goats. Front Genet. 2019;10:537. doi: 10.3389/fgene.2019.00537 31214253 PMC6558083

[19] Tarekegn GM, Khayatzadeh N, Liu B, Osama S, Haile A, Rischkowsky B, et al. Ethiopian indigenous goats offer insights into past and recent demographic dynamics and local adaptation in sub-Saharan African goats. Evol Appl. 2021;14(7):1716–31. doi: 10.1111/eva.13118 34295359 PMC8287980

[20] Serranito B, Taurisson-Mouret D, Harkat S, Laoun A, Ouchene-Khelifi N-A, Pompanon F, et al. Search for selection signatures related to Trypanosomosis tolerance in African goats. Front Genet. 2021;12:715732. doi: 10.3389/fgene.2021.715732 34413881 PMC8369930

[21] Purcell S, Neale B, Todd-Brown K, Thomas L, Ferreira MAR, Bender D, et al. PLINK: a tool set for whole-genome association and population-based linkage analyses. Am J Hum Genet. 2007;81(3):559–75. doi: 10.1086/519795 17701901 PMC1950838

[22] Alexander DH, Lange K. Enhancements to the ADMIXTURE algorithm for individual ancestry estimation. BMC Bioinformatics. 2011;12:246. doi: 10.1186/1471-2105-12-246 21682921 PMC3146885

[23] Alexander DH, Novembre J, Lange K. Fast model-based estimation of ancestry in unrelated individuals. Genome Res. 2009;19(9):1655–64. doi: 10.1101/gr.094052.109 19648217 PMC2752134

[24] Francis RM. pophelper: an R package and web app to analyse and visualize population structure. Mol Ecol Resour. 2017;17: 27–32. doi: 10.1111/1755-0998.12509 26850166

[25] Price AL, Patterson NJ, Plenge RM, Weinblatt ME, Shadick NA, Reich D. Principal components analysis corrects for stratification in genome-wide association studies. Nat Genet. 2006;38(8):904–9. doi: 10.1038/ng1847 16862161

[26] Patterson N, Price AL, Reich D. Population structure and eigenanalysis. PLoS Genet. 2006;2(12):e190. doi: 10.1371/journal.pgen.0020190 17194218 PMC1713260

[27] Wickham H. ggplot2: elegant graphics for data analysis. Springer-Verlag New York; 2016. Available from: https://ggplot2.tidyverse.org

[28] Weir BS, Cockerham CC. Estimating F-statistics for the analysis of population structure. Evolution. 1984;38(6):1358–70. doi: 10.1111/j.1558-5646.1984.tb05657.x 28563791

[29] Pickrell JK, Pritchard JK. Inference of population splits and mixtures from genome-wide allele frequency data. PLoS Genet. 2012;8(11):e1002967. doi: 10.1371/journal.pgen.1002967 23166502 PMC3499260

[30] Loh P-R, Lipson M, Patterson N, Moorjani P, Pickrell JK, Reich D, et al. Inferring admixture histories of human populations using linkage disequilibrium. Genetics. 2013;193(4):1233–54. doi: 10.1534/genetics.112.147330 23410830 PMC3606100

[31] Barbato M, Orozco-terWengel P, Tapio M, Bruford MW. SNeP: a tool to estimate trends in recent effective population size trajectories using genome-wide SNP data. Front Genet. 2015;6:109. doi: 10.3389/fgene.2015.00109 25852748 PMC4367434

[32] Sved JA. Linkage disequilibrium and homozygosity of chromosome segments in finite populations. Theor Popul Biol. 1971;2(2):125–41. doi: 10.1016/0040-5809(71)90011-6 5170716

[33] Ota T, Kimura M. Linkage disequilibrium between two segregating nucleotide sites under the steady flux of mutations in a finite population. Genetics. 1971;68(4):571–80. doi: 10.1093/genetics/68.4.571 5120656 PMC1212677

[34] Hayes BJ, Visscher PM, McPartlan HC, Goddard ME. Novel multilocus measure of linkage disequilibrium to estimate past effective population size. Genome Res. 2003;13(4):635–43. doi: 10.1101/gr.387103 12654718 PMC430161

[35] Corbin LJ, Liu AYH, Bishop SC, Woolliams JA. Estimation of historical effective population size using linkage disequilibria with marker data. J Anim Breed Genet. 2012;129(4):257–70. doi: 10.1111/j.1439-0388.2012.01003.x 22775258

[36] McQuillan R, Leutenegger A-L, Abdel-Rahman R, Franklin CS, Pericic M, Barac-Lauc L, et al. Runs of homozygosity in European populations. Am J Hum Genet. 2008;83: 658. doi: 10.1016/j.ajhg.2008.10.009 PMC2556426 18760389

[37] Meyermans R, Gorssen W, Buys N, Janssens S. How to study runs of homozygosity using PLINK? A guide for analyzing medium density SNP data in livestock and pet species. BMC Genomics. 2020;21(1):94. doi: 10.1186/s12864-020-6463-x 31996125 PMC6990544

[38] Curik I, Ferenčaković M, Sölkner J. Inbreeding and runs of homozygosity: a possible solution to an old problem. Livest Sci. 2014;166: 26–34. doi: 10.1016/j.livsci.2014.05.034

[39] Ceballos FC, Hazelhurst S, Ramsay M. Assessing runs of Homozygosity: a comparison of SNP Array and whole genome sequence low coverage data. BMC Genomics. 2018;19(1):106. doi: 10.1186/s12864-018-4489-0 29378520 PMC5789638

[40] Danecek P, Bonfield JK, Liddle J, Marshall J, Ohan V, Pollard MO, et al. Twelve years of SAMtools and BCFtools. Gigascience. 2021;10(2):giab008. doi: 10.1093/gigascience/giab008 33590861 PMC7931819

[41] Browning BL, Tian X, Zhou Y, Browning SR. Fast two-stage phasing of large-scale sequence data. Am J Hum Genet. 2021;108(10):1880–90. doi: 10.1016/j.ajhg.2021.08.005 34478634 PMC8551421

[42] Browning BL, Zhou Y, Browning SR. A one-penny imputed genome from next-generation reference panels. Am J Hum Genet. 2018;103(3):338–48. doi: 10.1016/j.ajhg.2018.07.015 30100085 PMC6128308

[43] Gautier M, Vitalis R. rehh: an R package to detect footprints of selection in genome-wide SNP data from haplotype structure. Bioinformatics. 2012;28(8):1176–7. doi: 10.1093/bioinformatics/bts115 22402612

[44] Gautier M, Klassmann A, Vitalis R. rehh 2.0: a reimplementation of the R package rehh to detect positive selection from haplotype structure. Mol Ecol Resour. 2017;17(1):78–90. doi: 10.1111/1755-0998.12634 27863062

[45] Szpiech ZA, Hernandez RD. selscan: an efficient multithreaded program to perform EHH-based scans for positive selection. Mol Biol Evol. 2014;31(10):2824–7. doi: 10.1093/molbev/msu211 25015648 PMC4166924

[46] Szpiech ZA. selscan 2.0: scanning for sweeps in unphased data. Bioinformatics. 2024;40(1):btae006. doi: 10.1093/bioinformatics/btae006 38180866 PMC10789311

[47] Garud NR, Messer PW, Buzbas EO, Petrov DA. Recent selective sweeps in North American *Drosophila melanogaster* show signatures of soft sweeps. PLoS Genet. 2015;11(2):e1005004. doi: 10.1371/journal.pgen.1005004 25706129 PMC4338236

[48] Rubin C-J, Zody MC, Eriksson J, Meadows JRS, Sherwood E, Webster MT, et al. Whole-genome resequencing reveals loci under selection during chicken domestication. Nature. 2010;464(7288):587–91. doi: 10.1038/nature08832 20220755

[49] Verity R, Collins C, Card DC, Schaal SM, Wang L, Lotterhos KE. minotaur: a platform for the analysis and visualization of multivariate results from genome scans with R Shiny. Mol Ecol Resour. 2017;17(1):33–43. doi: 10.1111/1755-0998.12579 27473028

[50] Ma Y, Ding X, Qanbari S, Weigend S, Zhang Q, Simianer H. Properties of different selection signature statistics and a new strategy for combining them. Heredity (Edinb). 2015;115(5):426–36. doi: 10.1038/hdy.2015.42 25990878 PMC4611237

[51] Benjamini Y, Hochberg Y. Controlling the false discovery rate: a practical and powerful approach to multiple testing. J R Stat Soc Ser B Stat Methodol. 1995;57(1):289–300. doi: 10.1111/j.2517-6161.1995.tb02031.x

[52] Yates AD, Achuthan P, Akanni W, Allen J, Allen J, Alvarez-Jarreta J, et al. Ensembl 2020. Nucleic Acids Res. 2020;48: D682–D688. doi: 10.1093/nar/gkz966 PMC7145704 31691826

[53] Durinck S, Spellman PT, Birney E, Huber W. Mapping identifiers for the integration of genomic datasets with the R/Bioconductor package biomaRt. Nat Protoc. 2009;4(8):1184–91. doi: 10.1038/nprot.2009.97 19617889 PMC3159387

[54] Kolberg L, Raudvere U, Kuzmin I, Vilo J, Peterson H. gprofiler2 -- an R package for gene list functional enrichment analysis and namespace conversion toolset g:Profiler. F1000Res. 2020;9:ELIXIR-709. doi: 10.12688/f1000research.24956.2 33564394 PMC7859841

[55] Csardi G, Nepusz T. The igraph software package for complex network research. InterJournal, Complex Syst; 2006. Available from: http://igraph.org/

[56] Csárdi G, Nepusz T, Traag V, Horvát S, Zanini F, Noom D, et al. igraph: network analysis and visualization in R. https://CRAN.R-project.org/package=igraph. R package version 2.1.1; 2025.

[57] Blench R. Traditional livestock breeds: geographical distribution and dynamics in relations to the ecology of West Africa. Overseas Dev Inst Portl House Stag Place London, SW1E 5DP. 1999; Working Paper 122. Pages: 1–69.

[58] Murital I, Afolayan O, Bemji MN, Dadi O, Landi V, Martínez A, et al. Genetic diversity and population structure of Nigerian indigenous goat using DNA microsatellite markers. Arch Zootec. 2015;64: 93–8. doi: 10.21071/az.v64i246.382

[59] Berihulay H, Islam R, Jiang L, Ma Y. Genome-wide linkage disequilibrium and the extent of effective population sizes in six Chinese goat populations using a 50K single nucleotide polymorphism panel. Animals (Basel). 2019;9(6):350. doi: 10.3390/ani9060350 31200540 PMC6617254

[60] Zhao Q, Huang C, Chen Q, Su Y, Zhang Y, Wang R, et al. Genomic inbreeding and runs of homozygosity analysis of cashmere goat. Animals. 2024;14. doi: 10.3390/ani14081246 PMC11047310 38672394

[61] Frankham R, Bradshaw CJA, Brook BW. Genetics in conservation management: revised recommendations for the 50/500 rules, Red List criteria and population viability analyses. Biol Conserv. 2014;170: 56–63. doi: 10.1016/j.biocon.2013.12.036

[62] Waineina RW, Okeno TO, Ilatsia ED, Ngeno K. Selection signature analyses revealed genes associated with adaptation, production, and reproduction in selected goat breeds in Kenya. Front Genet. 2022;13. doi: 10.3389/fgene.2022.858923 PMC9068939 35528543

[63] Hynes RO. Integrins: bidirectional, allosteric signaling machines. Cell. 2002;110(6):673–87. doi: 10.1016/s0092-8674(02)00971-6 12297042

[64] Stewart PL, Nemerow GR. Cell integrins: commonly used receptors for diverse viral pathogens. Trends Microbiol. 2007;15(11):500–7. doi: 10.1016/j.tim.2007.10.001 17988871

[65] Fakae BB, Chiejina SN, Behnke JM, Ezeokonkwo RC, Nnadi PA, Onyenwe WI, et al. The response of Nigerian West African Dwarf goats to experimental infections with Haemonchus contortus. Res Vet Sci. 1999;66(2):147–58. doi: 10.1053/rvsc.1998.0262 10208893

[66] Zetterberg H. Neurofilament light: a dynamic cross-disease fluid biomarker for neurodegeneration. Neuron. 2016;91: 1–3. doi: 10.1016/j.neuron.2016.06.030 27387643

[67] Sainio MT, Rasila T, Molchanova SM, Järvilehto J, Torregrosa-Muñumer R, Harjuhaahto S, et al. Neurofilament light regulates axon caliber, synaptic activity, and organelle trafficking in cultured human motor neurons. Front Cell Dev Biol. 2022;9:820105. doi: 10.3389/fcell.2021.820105 35237613 PMC8883324

[68] Niimura Y, Nei M. Extensive gains and losses of olfactory receptor genes in mammalian evolution. PLoS One. 2007;2(8):e708. doi: 10.1371/journal.pone.0000708 17684554 PMC1933591

[69] Bertolini F, Servin B, Talenti A, Rochat E, Kim ES, Oget C, et al. Signatures of selection and environmental adaptation across the goat genome post-domestication. Genet Sel Evol. 2018;50(1):57. doi: 10.1186/s12711-018-0421-y 30449276 PMC6240954

[70] Muriuki C, Bush SJ, Salavati M, McCulloch MEB, Lisowski ZM, Agaba M, et al. A mini-atlas of gene expression for the domestic goat (*Capra hircus*). Front Genet. 2019;10:1080. doi: 10.3389/fgene.2019.01080 31749840 PMC6844187

[71] Fu W, Wang R, Nanaei HA, Wang J, Hu D, Jiang Y. RGD v2.0: a major update of the ruminant functional and evolutionary genomics database. Nucleic Acids Res. 2022;50(D1):D1091–9. doi: 10.1093/nar/gkab887 34643708 PMC8728256

[72] Nielsen R, Hellmann I, Hubisz M, Bustamante C, Clark AG. Recent and ongoing selection in the human genome. Nat Rev Genet. 2007;8(11):857–68. doi: 10.1038/nrg2187 17943193 PMC2933187

