# Supplementary figures and images for: Population genomics of Nigerian goat breeds and neighbouring populations in the West Africa–Cameroon transboundary livestock corridor
Source: PLoS One. 2026 Jul 27;21(7):e0354294. doi: 10.1371/journal.pone.0354294 (PMC13405285; doi:10.1371/journal.pone.0354294)

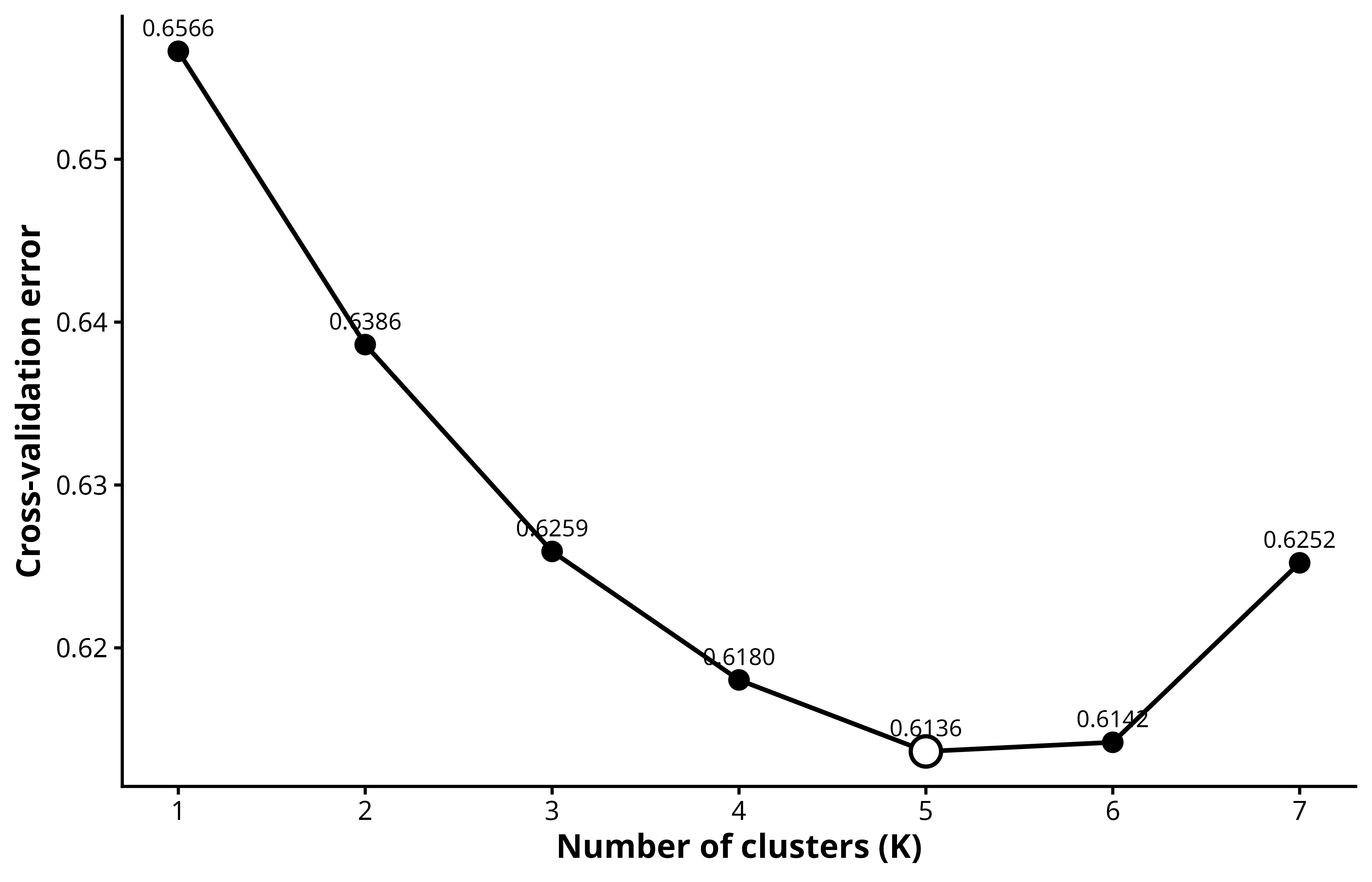

Supplement: S1 Fig — (TIFF) [file pone.0354294.s001.tiff]

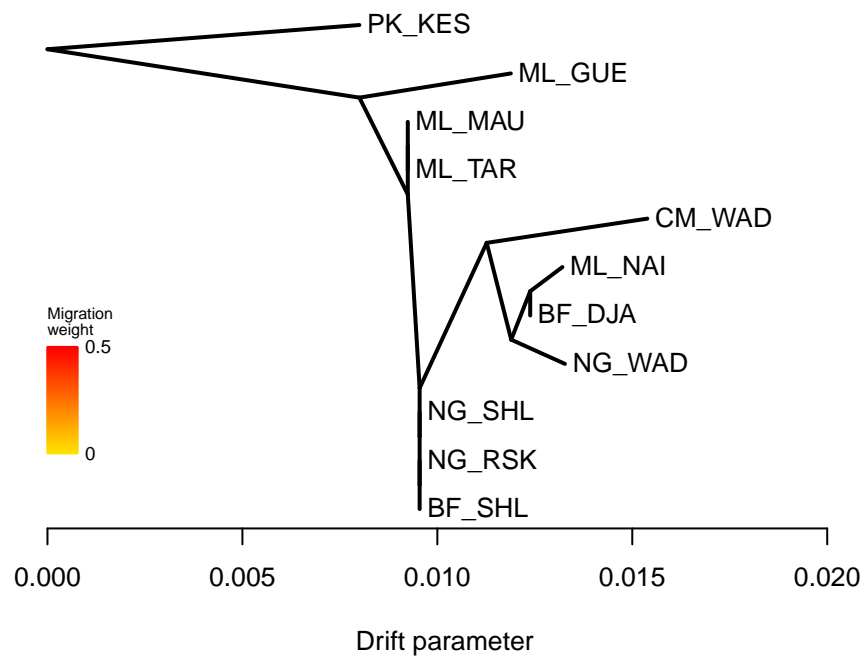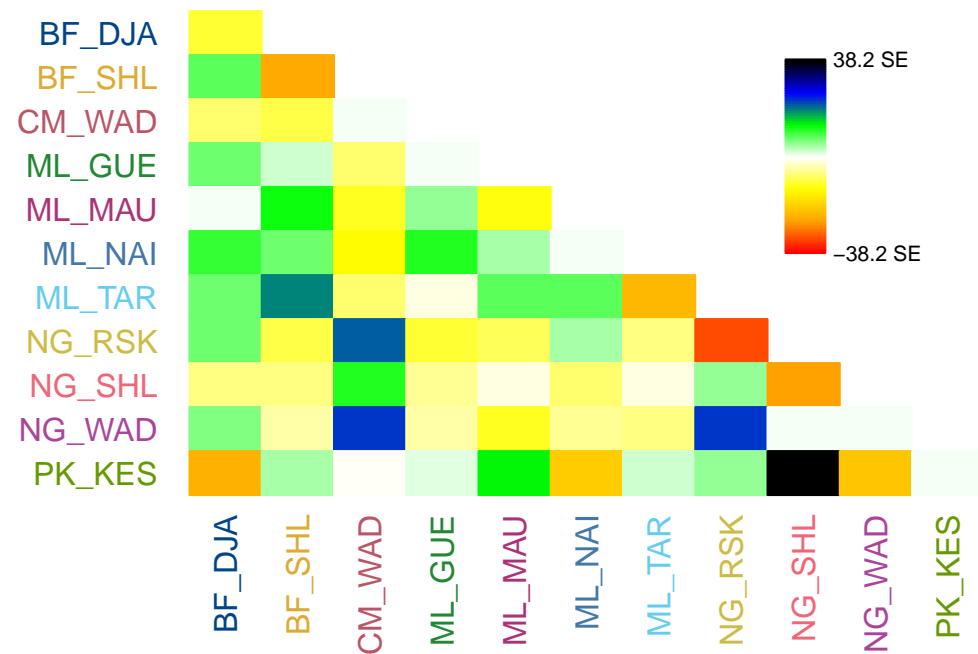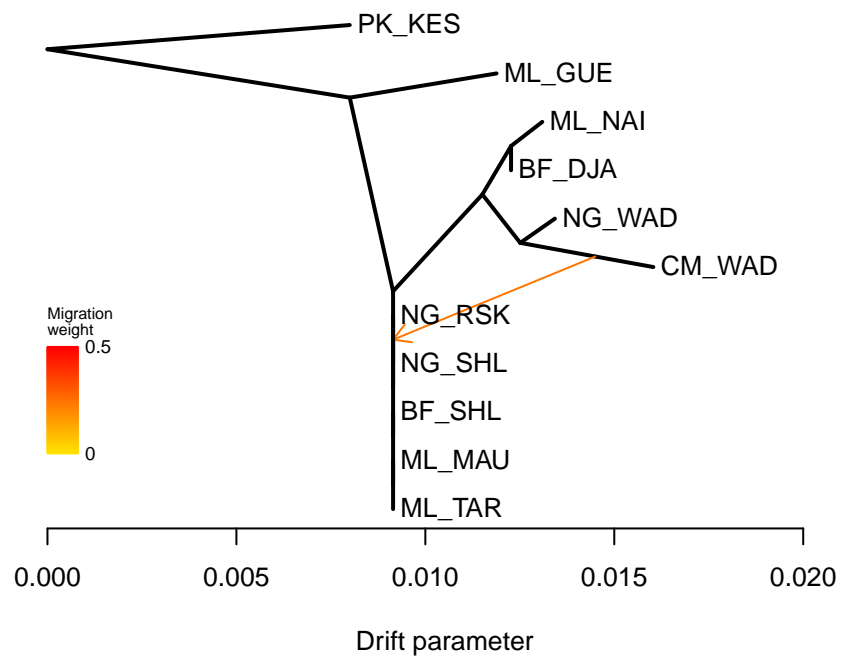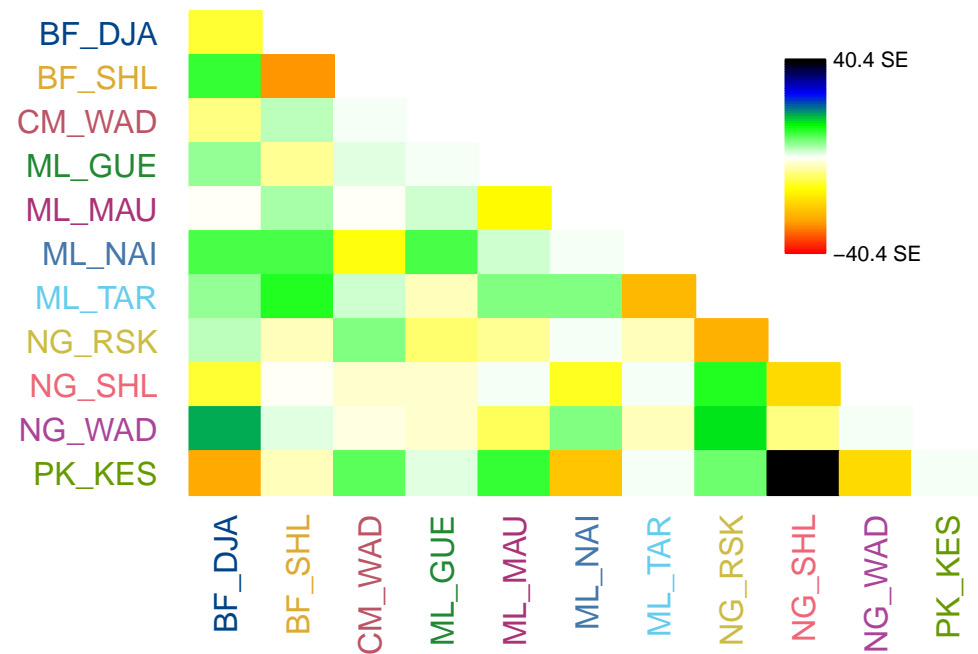

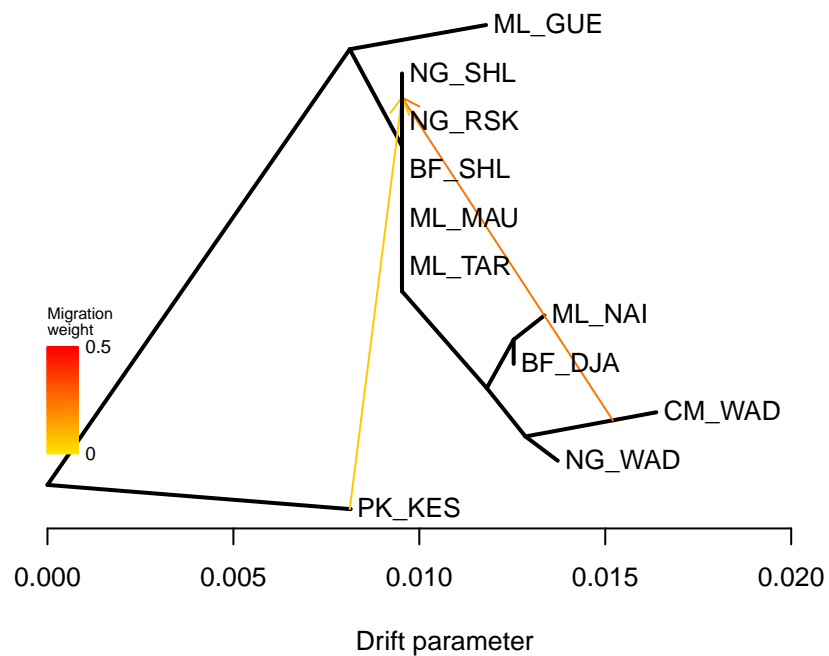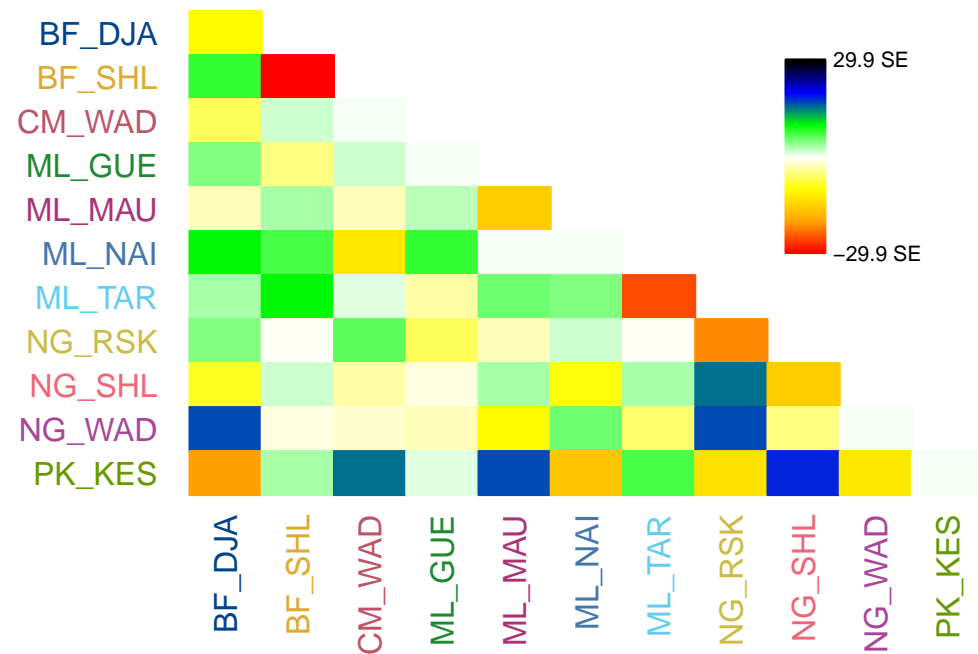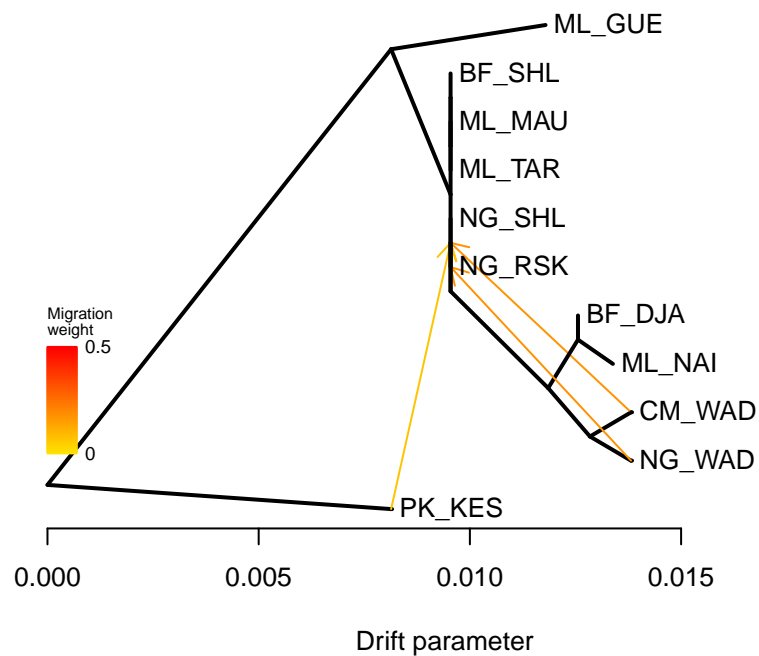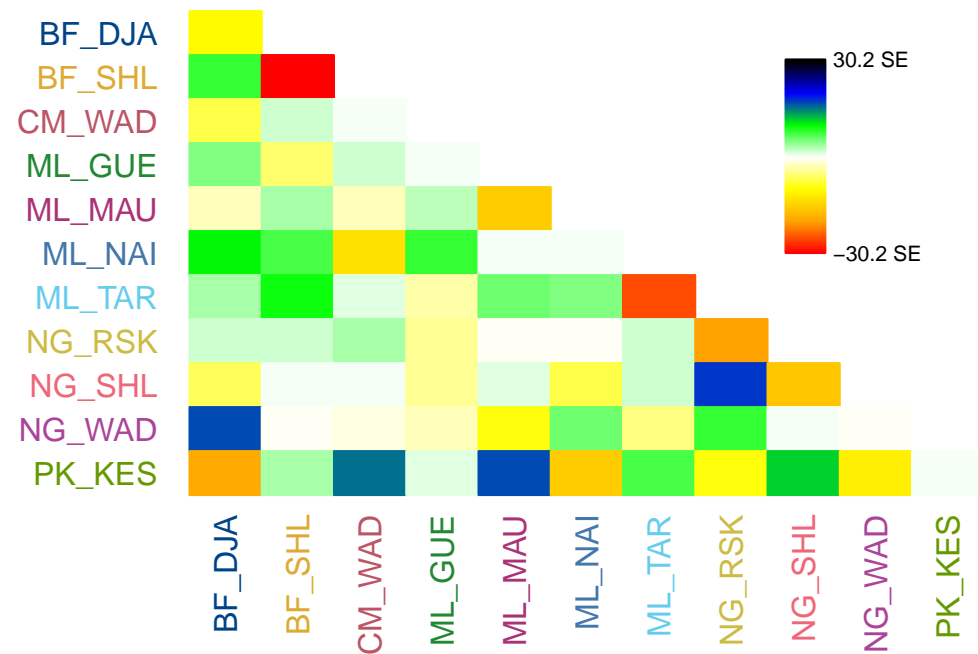

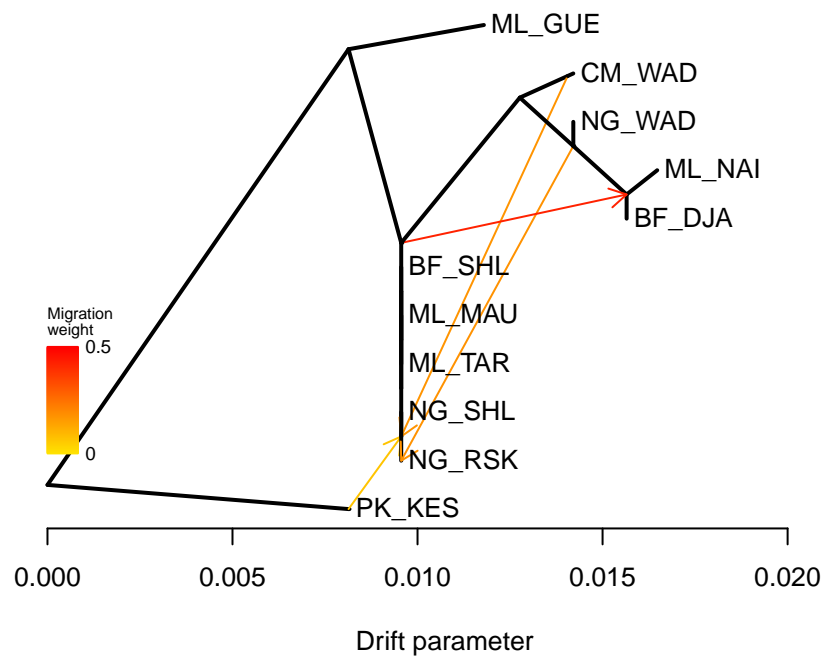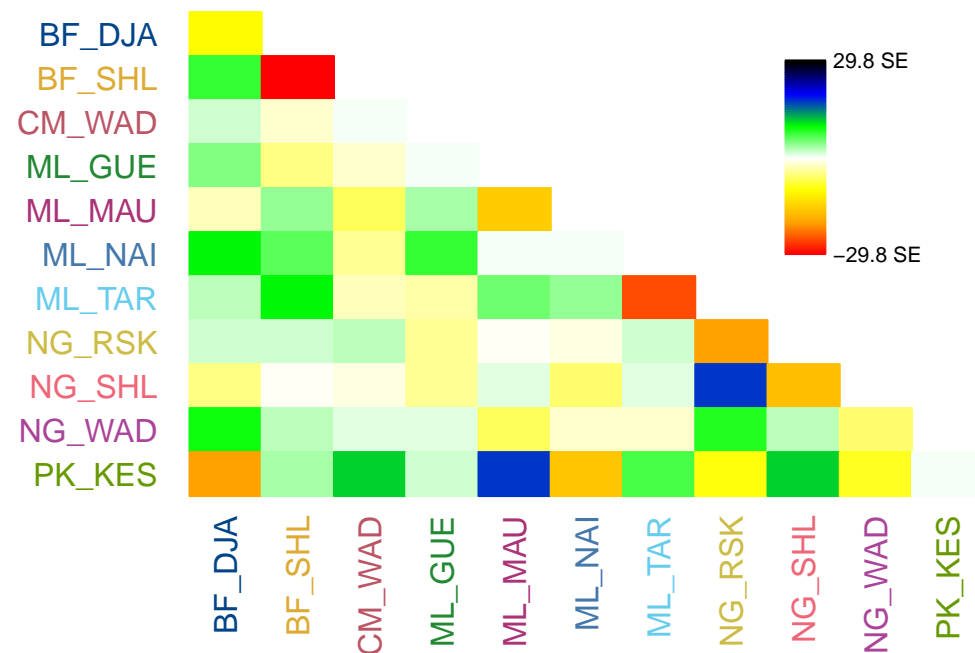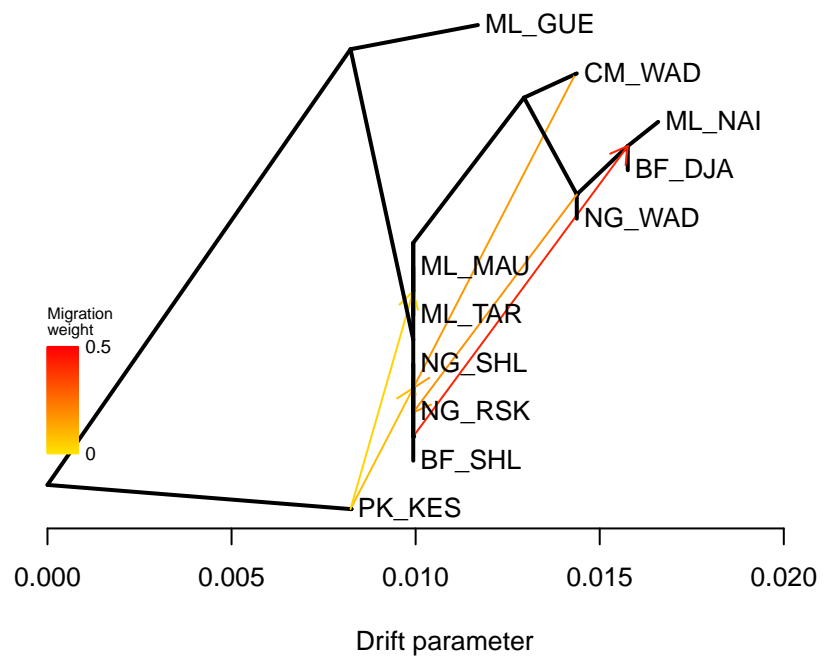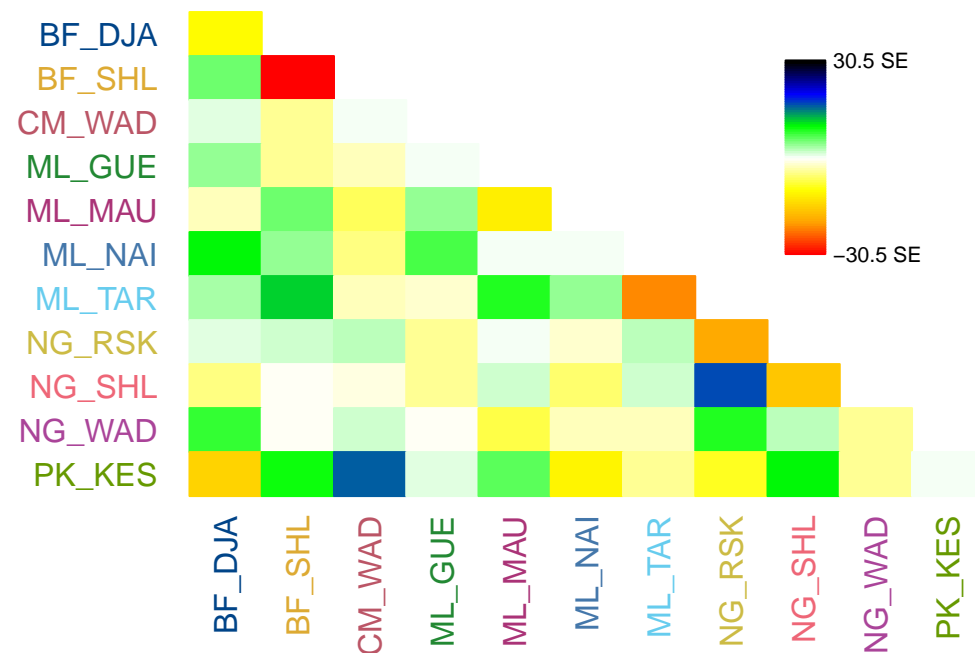

Supplement: S1 File — (PDF) [file pone.0354294.s008.pdf]
