# Supplementary material for: Population genomics of Nigerian goat breeds and neighbouring populations in the West Africa–Cameroon transboundary livestock corridor
Source: PLoS One. 2026 Jul 27;21(7):e0354294. doi: 10.1371/journal.pone.0354294 (PMC13405285; doi:10.1371/journal.pone.0354294)
Supplement: Attachment — Submitted filename: Response to Reviewers.docx [file pone.0354294.s010.docx]

Dear Editor and Esteemed Reviewers,

Thank you very much for your thoughtful and constructive feedback on our manuscript, “Population genomics of Nigerian goat breeds and neighbouring populations in the West Africa–Cameroon transboundary livestock corridor.” We greatly appreciate the time and care invested in reviewing our work.

In this minor revision, we have addressed the reviewers’ remaining comments by clarifying key methodological and interpretative points, including the definition of transboundary genomic backgrounds, the interpretation of ALDER admixture dates, the reporting of ROH results, and the discussion of SNP-chip ascertainment bias. We also made minor textual and formatting corrections where needed.

Detailed responses to each comment are provided in the attached point-by-point response table. We believe the revised manuscript now satisfactorily addresses the reviewers’ concerns.

Thank you once again for your valuable feedback and consideration.

Kind regards,

O.M. Akinsola, A.A. Musa, and co-authors

| **No** | **Comment** | **Author’s response** |
| --- | --- | --- |
| **Reviewer 1** | | |
| **1** | Some sample sizes include less than 20 animals. Is this sufficient number to perform a search for signatures of selection? The justification id required. | We agree that modest sample sizes limit power for formal, population-specific confirmation of selective sweeps. However, there is no universal sample-size threshold for selection scans as power depends on selection strength and timing, allele frequency, LD structure, demography and marker density. We therefore used DCMS for candidate region prioritisation rather than formal confirmation. This has been clarified in the limitations section.  See lines 656–659 in the revised manuscript. |
| **2** | L200-201 Please provide clarification why these criteria were chosen (2 missing calls and 0 heterozygotes)? | Two missing calls were allowed to prevent isolated missing genotypes from fragmenting ROH, whereas zero heterozygotes were allowed to keep ROH detection conservative in medium-density SNP data. See lines 215–217 in the revised manuscript. |
| **3** | L208 -213 This paragraph looks like as data processing of WGS data and not of SNP-genotyping data. Because SNP-genotyping data is biallelic and do not require splitting multiallelic sites. | We thank the reviewer for noting this. The data were SNP-chip genotypes, and standard SNP-chip QC had already restricted the dataset to autosomal biallelic SNPs before the haplotype analyses. The BCFtools step was used only for VCF-format checking after chromosome-wise VCF export, including removal of duplicate records before phasing. We have removed the phrase “split multiallelic sites” to avoid any impression that WGS variant processing was performed. See lines 226–227 in the revised manuscript. |
| **4** | Why the authors did chose predominantly methods based on haplotypes analysis? | We have included the rationale in the Methods. Haplotype-based statistics were the predominant methods because recent or incomplete sweeps can generate extended haplotype homozygosity even when between-population allele-frequency differentiation remains modest. However, the DCMS framework was not exclusively haplotype-based, as ZHp was also included to identify windows with unusually low pooled heterozygosity. See lines 261–264 in the revised manuscript. |
| **5** | The description of the ROH statistics is missing in the Results. | We have added a results paragraph summarising the ROH statistics from S6 Table. The revised text now reports the range of mean FROH across populations, identifies populations with the highest and lowest ROH burden, and describes the contribution of long ROH segments to recent autozygosity. See lines 420–426 in the revised manuscript. |
| **6** | It would be more informative if the authors add the grouping of studied populations according to the climate zone to the Figure 1. | We thank the reviewer for this suggestion. We did not add climate-zone grouping to Figure 1 because climate zone was not analysed as a discrete variable. Figure 1 shows ADMIXTURE ancestry proportions by sampled population, and adding a post hoc climate-zone grouping could confuse readers by implying a formal climate-based classification. We therefore retained the population-based ordering. |
| **7** | The outgroup PK-KES is missing in Figure 6 but it is presented in the description of this figure. | Thank you for identifying this. The mention of PK_KES was an error and has been deleted. See line 428 in the revised manuscript. |
| **Reviewer 2** | | |
| **8** | This is a good paper with clear objectives, robust analyses, and a meaningful contribution to the understanding of goat population genomics in West Africa. The transboundary perspective is particularly valuable and aligns well with real livestock mobility patterns in the region. The paper would benefit from additional clarity in key methodological and interpretative sections, as detailed below. | We thank the reviewer for the positive assessment of the manuscript and for recognising the value of the transboundary perspective. We have addressed the requested clarifications in the revised manuscript, particularly regarding the definition of transboundary genomic backgrounds, ALDER interpretation, and biological interpretation of the DCMS results. See details below. |
| **9** | Definition and justification of “transboundary genomic backgrounds”: this concept is central to the paper, but is not formally defined. Please clarify: the criteria used to delineate these backgrounds (ADMIXTURE? PCA? FST? TreeMix?); whether they represent discrete clusters or gradients; how stable they are across alternative K values in ADMIXTURE. | We have now formally defined “transboundary genomic backgrounds” in the Introduction as broad genomic affinities shared across national borders, rather than closed national breed units. See page 6, lines 104–107. The Results then describe how these backgrounds were inferred from ADMIXTURE and supported by PCA, pairwise FST, and TreeMix. See pages 15–18. We also clarify that these backgrounds represent broad genomic affinities and gradients, not strict discrete clusters; ADMIXTURE patterns across K=2–5 are shown in Figure 1, with model choice supported by S1 Fig. |
| **10** | Interpretation of ALDER admixture dates: the ALDER section requires more clearness. Please clarify: which curves were excluded and why; how you distinguish admixture related LD from LD due to demographic contraction; whether the very recent dates (26–38 years) are consistent with known patterns of cross border livestock trade. Including representative ALDER decay plots as supplementary figures would strengthen the analysis. | We thank the reviewer for this helpful comment. We have clarified the ALDER Methods and Results. The revised text now states how one-reference weighted-LD fits were extracted for each target–reference combination, how the 𝑑min sensitivity grid was used, and how signals were classified as stable, consistent, fragile or none. We also clarified that target–reference combinations with no significant LD-decay fit are reported in S2 Table but not plotted in Figure 5, whereas fragile signals are retained but interpreted cautiously. Finally, we clarified that ALDER dates are LD-decay proxy dates rather than exact historical events and should be interpreted together with the population-structure and gene-flow results. See lines 180-196 and 384-399 in the revised manuscript. |
| **11** | Selection signatures (DCMS): need for deeper biological interpretation. The DCMS results are interesting, but underdeveloped. Additional information: listing key candidate regions and genes; discussing plausible adaptive functions in relation to ecology (Sahel vs humid belt); clarifying whether signals are shared or background specific. This will help readers understand the biological relevance of the findings. | We have clarified the DCMS interpretation in the Results and Discussion. The revised Results now distinguish background-aligned signals from cross-background corridor-level candidate windows. The revised Discussion also states explicitly that DCMS results are interpreted at two levels: shared/background-aligned genomic windows and functional themes that generate phenotype-facing hypotheses. We retained the detailed biological interpretation by genomic background, including adhesion/integrin and translation quality-control themes in the Djallonké/WAD background, neuronal/neuroendocrine themes in ML_GUE, and olfactory receptor/chemosensory themes in the Sahel–Sudan background. See lines 451-454 and 593-595 in the revised manuscript. |
| **12** | Abstract: consider rounding highly specific temporal estimates (e.g., “1,016 years”). Clarify that ALDER provides proxy dates based on LD decay. | We have revised the Abstract to avoid over-precise temporal wording and to clarify the nature of the admixture-timing estimates. The revised text now describes the dates as approximate estimates inferred from linkage-disequilibrium decay, and the temporal estimates have been rounded, for example from ~26–38 years to ~30–40 years and from ~160–1,016 years to ~160–1,000 years. See lines 38-41. |
| **13** | Introduction: consider adding recent references on goat genomics in Central Africa and on pastoral mobility/trade networks. | We have strengthened the Introduction by making the Central African goat genomics context more direct, particularly genome-wide evidence for the differentiation of Cameroon goats within broader African and Asian comparative panels. We also added references on drought adaptation, trade histories and contemporary transhumance to support the statement that Sahelian pastoral zones, savanna trade corridors and humid-belt smallholder systems are connected by livestock mobility and exchange. See lines 70-72. |
| **14** | Methods: Specify the exact ARS1 assembly version. Clarify whether FST was computed on the LD pruned or full SNP set. Provide window size and parameters used for DCMS. | The genotype data were analysed as provided in the public AdaptMap GoatSNP50 repository, and we did not perform additional SNP remapping. Because the source dataset reports ARS1 coordinates and does not provide a more specific alternative version for the PLINK files used here, we retained the assembly description as ARS1 rather than adding an unsupported version label. We have clarified that pairwise 𝐹ST was computed using the dense unrelated post-QC marker set (209 individuals; 46,431 autosomal SNPs), not the LD-pruned marker set (lines 162-163). The DCMS Methods already specify the 500-kb non-overlapping autosomal windows, the five statistics used, empirical rank-based p-values with predefined tail directions, correlation-aware weighting, and FDR control at 5%; these details have been retained. |
| **15** | Results: PCA figures would benefit from more contrasting colors. For ADMIXTURE, indicate variance across replicates (if applicable). In TreeMix, specify the final number of migration edges selected. | We reviewed the PCA figure and retained the current colour/shape scheme because colour encodes country/source region and point shape distinguishes breed/population, which preserves both regional and population-level information. ADMIXTURE replicate variance is not applicable because replicate runs were not performed. For TreeMix, the Results already specify that graphs were fitted with 0–6 migration edges and that m = 4 was retained for interpretation based on likelihood and residual-covariance diagnostics; the four inferred migration edges are also listed in the Results and shown in Figure 4. |
| **16** | Discussion: add a short paragraph on the potential influence of SNP chip ascertainment bias on selection scans and demographic inference. | We have expanded the methodological limitations section to address the potential influence of SNP-chip ascertainment bias on both selection scans and demographic inference. The revised text now explains that ascertainment bias can affect allele-frequency distributions, population differentiation and linkage-disequilibrium patterns, may reduce power for detecting population-specific or poorly tagged variants, and can influence LD-based demographic inference. We therefore interpret the selection-scan and LD-based Ne results comparatively and cautiously. See lines 648-653 |
